# Hydrogen Atomic Positions of O–H···O Hydrogen Bonds in Solution and in the Solid State: The Synergy of Quantum Chemical Calculations with ^1^H-NMR Chemical Shifts and X-ray Diffraction Methods

**DOI:** 10.3390/molecules22030415

**Published:** 2017-03-07

**Authors:** Michael G. Siskos, M. Iqbal Choudhary, Ioannis P. Gerothanassis

**Affiliations:** 1 Section of Organic Chemistry & Biochemistry, Department of Chemistry, University of Ioannina, Ioannina GR-45110, Greece; msiskos@cc.uoi.gr; 2 H.E.J. Research Institute of Chemistry, International Center for Biological and Chemical Sciences, University of Karachi, Karachi 75270, Pakistan; iqbalhej@yahoo.com

**Keywords:** chemical shifts, hydrogen bonding, DFT, X-ray diffraction, NMR

## Abstract

The exact knowledge of hydrogen atomic positions of O–H···O hydrogen bonds in solution and in the solid state has been a major challenge in structural and physical organic chemistry. The objective of this review article is to summarize recent developments in the refinement of labile hydrogen positions with the use of: (i) density functional theory (DFT) calculations after a structure has been determined by X-ray from single crystals or from powders; (ii) ^1^H-NMR chemical shifts as constraints in DFT calculations, and (iii) use of root-mean-square deviation between experimentally determined and DFT calculated ^1^H-NMR chemical shifts considering the great sensitivity of ^1^H-NMR shielding to hydrogen bonding properties.

## 1. Introduction

Hydrogen bonding is a fundamental aspect in the determination of three-dimensional structures, reactivity, and functions of biological macromolecules, for encoding genetic information, in crystal engineering and in material sciences [1,2,3,4,5,6,7,8]. Although detection of hydrogen bonds remains an area of active research and the characterization of hydrogen bond interactions has been the subject of numerous experimental and theoretical studies, the detailed understanding of the nature of hydrogen bonding and its structural, energetic, and dynamic properties are still limited. This is due to the fact that the strength of the hydrogen bond depends on several factors, such as the X–H and H···Y lengths, the X–H···Y–Z dihedral angle, the nature of the microenvironment [8,9], the p*K*_a_/p*K*_b_ values of the participating components [10,11,12], and molecular electrostatic potential surfaces [13,14].

X-ray and neutron diffraction of single crystals and, in some cases, of powder samples are the most widespread and popular methods for investigating hydrogen bonding interactions in the solid state, often in conjunction with structural indicators such as bond lengths and bond angles [15,16]. However, the unequivocal determination of hydrogen atomic positions is not straightforward particularly with systems exhibiting proton disorder [1,7,17]. Furthermore, since the X-ray diffraction experiments determine electron density distributions, in a covalent X–H bond with an electronegative atom X, the average position of the electron charge density of the hydrogen atom is displaced towards the X atom. As a result, numerous X-ray structures yielded unrealistic OH bond lengths (~0.8–0.9 Å, compared to a typical value of 1.0 Å), ambiguous conclusions with respect to the molecular ionization states (resulted from proton transfer processes), and the hydrogen bonding network in the crystal lattice. Neutron diffraction, on the other hand, locates the nuclei because neutrons are scattered by nuclei [7]. The results of the two techniques for hydrogen atoms often differ by more than 0.1 Å [18]. It has become a practice in the analysis of X-ray diffraction results to normalize the X–H bonds [19] by increasing the position of hydrogen atoms by a relaxed amount [20] or restraint to a common value from highly accurate neutron diffraction experiments [21]. In addition, significant efforts have been made in assigning the positions of hydrogen atoms from X-ray diffraction experiments with the use of quantum chemical methods [22,23,24].

NMR spectroscopy is among the primary methods for investigating hydrogen bonding interactions both in solution and in the solid states [25]. The existence of hydrogen bond is inferred from several NMR methods, such as chemical shifts [26,27,28,29,30,31,32,33], temperature dependence of chemical shifts [32,33], solvent accessibility [34], the NOE phenomenon [35,36], direct spin-spin scalar coupling between nuclei on both sides of the hydrogen bonds [37,38], isotope effects [4,9,39], REDOR experiments [40], and NMR dipolar coupling in the solid states [41,42]. Developments in quantum chemical methods for calculating NMR chemical shifts [43,44,45] have led to an increasing number of studies which focus on the assignment or reassignment of individual protons and carbons [40], including hydrogen bonding effects [46,47,48], in the elucidation of chemical structures [45] and in the refinement of labile hydrogen positions [49,50]. Such calculations have also played an important role in the new field of NMR crystallography where NMR spectroscopy is combined with X-ray diffraction to aid structural information [51,52,53].

In the present review, we will summarize recent developments in the determination of labile hydrogen atomic positions in O–H···O hydrogen bonds with the combined use of DFT calculations after a structure has been determined by X-ray from single crystals or from powders, and the use of root-mean-square deviation of experimental ^1^H-NMR chemical shifts with DFT calculated chemical shifts. Finally, we will comment on the future development of this field.

## 2. Accurate Hydrogen Atom Positions with the Combined Use of X-ray and Quantum Chemical Methods

The aim of this integrated methodology is the production of a structural model with DFT optimization of the crystalographically determined structure. Allan and Clark [22] determined the high pressure crystal structures of ethanol and acetic acid including the positions of the hydrogen atoms using a combination of single crystal X-ray diffraction techniques and DFT calculations within the generalized gradient approximation for the exchange and correlation potential [54]. The experimentally determined unit-cell parameters and the heavy atom positions were used as an input geometry, and approximate positions for the hydrogen atoms were used. The valence electron wave functions were expanded in a plane-wave basis to an energy cutoff of 700 eV. The fully relaxed structural parameters including unit-cell parameters and the position of the hydrogen atoms were determined. Additional DFT calculations were also performed without constraining the structures to any space group which demonstrated that the symmetry of the structure was preserved.

Florence et al. [55] utilized geometry optimization using DFT in CASTEP code [56]. It is an effective method to locate hydrogen atom positions which provides a more accurate description of molecular conformation and intermolecular interactions from powder diffraction data than global optimization and retrieved refinement.

Deringer et al. [24] presented a comprehensive investigation of a large number of structures from the Cambridge Structural Database and compared computationally optimized X–Y bond lengths with neutron diffraction data, whenever available. DFT optimizations were performed using the generalized gradient approximation as parameterized by Perdew, Burke and Ernzerhof [57]. This is a method of choice in many investigations of this type. Since in the X-ray structures the hydrogen atomic positions are not optimum, three protocols for geometry optimization can be performed: (i) optimization of the hydrogen positions within a unit cell; (ii) optimization of all atomic positions within a unit cell and (iii) optimization of all atomic positions and unit cell vectors. The selective relaxation of only the hydrogen atoms was taken into account since it was demonstrated that both full relaxation of all the atomic positions and the selective relaxation of the hydrogen atoms resulted in, practically, the same values. Furthermore, the computation time for selective relaxation was substantially reduced (~300%) with respect to the time required for full relaxation of all the atomic positions.

**Figure 1:**
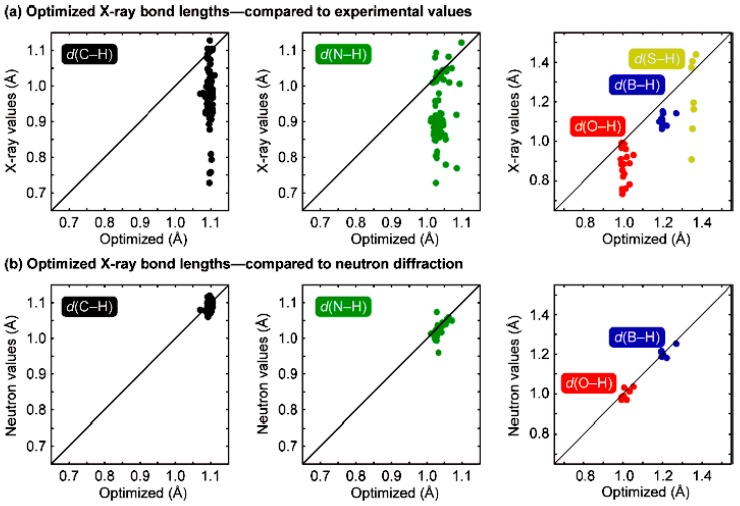
Bond lengths of the investigated structures. (**a**) X-ray determined values (vertical axis) plotted against quantum-chemically optimized ones (horizontal axis); (**b**) Quantum-chemically optimized values (horizontal axis) vs. the available neutron diffraction data. Covalent C–H (left), N–H (middle), O–H, S–H, and B–H (right) bond lengths are considered. Reprinted, with permission, from [24]. Copyright 2012, by The American Chemical Society.

**Figure 2:**
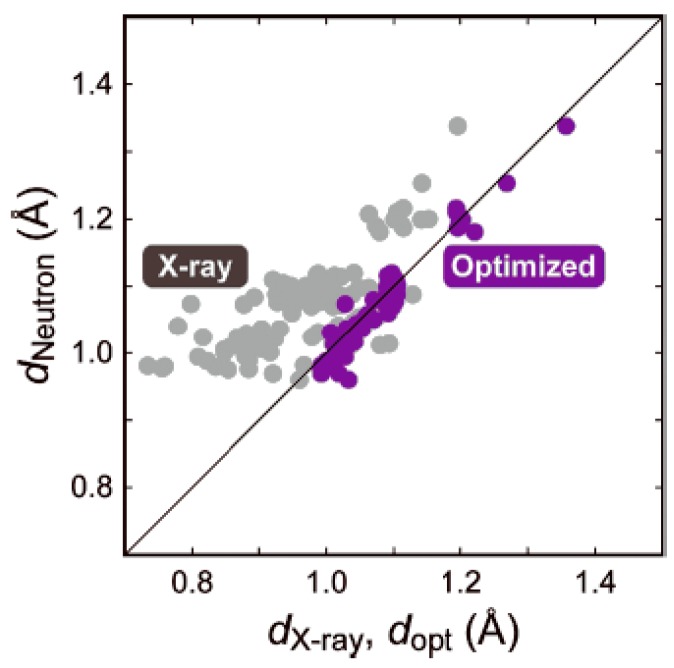
Neutron diffraction X–H bond length data compared both to X-ray-data (light gray circles) and quantum-chemically optimized values (purple circles). Values for X = C, N, O, S, B are given. Reprinted, with permission, from [24]. Copyright 2012, by The American Chemical Society.

Figure 1a illustrates a plot of the X-ray structural data (vertical axis) vs. the quantum chemically optimized values (horizontal axis) for C–H, N–H, O–H, B–H, and S–H bonds [19]. In all cases a wide range of values was observed in the X-ray structures. On the contrary, after quantum chemical optimization of the X-ray structures, a very good agreement with the neutron diffraction data was observed (Figure 1b). The S–H data were omitted from the correlation due to ambiguities in the S–H bond lengths of the neutron diffraction experiments. Figure 2 illustrates the neutron diffraction data of all the X–H bond lengths vs. the X-ray structural data (light gray circles) and those obtained after quantum chemical optimization (purple circles). Again the quantum chemically optimized structures were in very good agreement with those obtained from neutron diffraction experiments [24]. It should be emphasized, however, that DFT calculations typically do not include temperature effects, therefore, they represent the molecular system at absolute zero. Since X-ray diffraction experiments are usually performed between room temperature and cryogenic temperatures, the experimental unit cell volumes and atomic positions may differ from those resulting from DFT calculations.

**Scheme 1:**
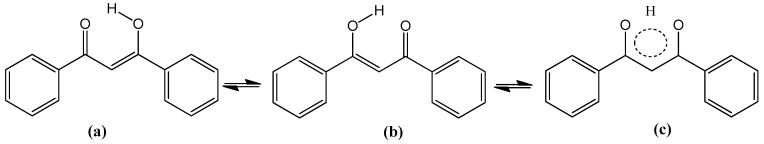
Tautomeric equilibrium of dibenzoylmethane.

**Figure 3:**
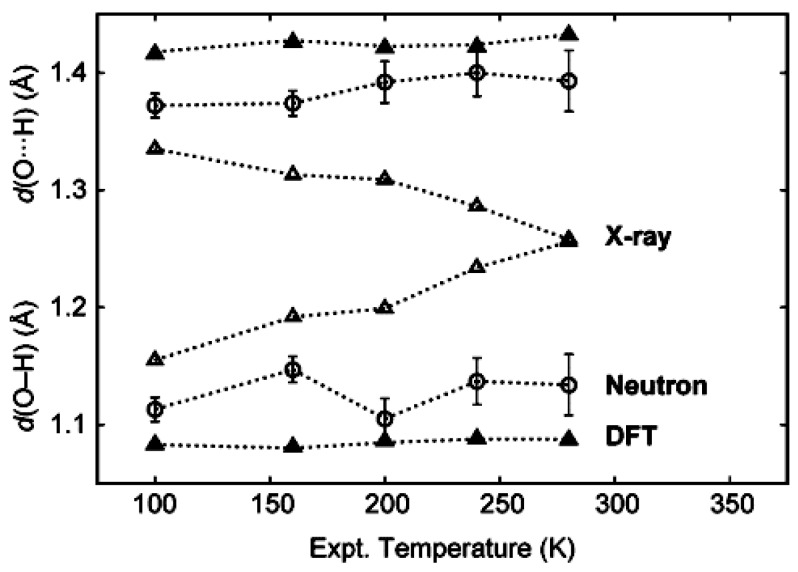
Effects of temperature on the O–H and O···H distances of dibenzoylmethane. X-ray (open triangles) and neutron diffraction (open circles) data [61] are plotted along with DFT optimized O–H and O···H bond lengths (filled triangles) [24]. Error bars are plotted for the neutron-derived data, while those for X-ray data have been left out for clarity, and dotted lines serve only as guides to the eye [56]. Reprinted, with permission, from [24]. Copyright 2012, by The American Chemical Society.

The case of dibenzoylmethane is of particular interest due to keto-enol tautomeric equilibrium which is characterized with a strong intramolecular hydrogen bond interaction. The shape of the low barrier O–H···O potential function either a double minimum potential corresponding to two tautomeric forms (a) and (b), or a single minimum symmetrical one in a strongly delocalized system (c) (Scheme 1), has been the subject of extensive studies [58,59,60]. The O–H and O···H distances, measured by neutron diffraction [61], were found to be unchanged over the temperature range of 100 to 280 K (Figure 3). The X-ray diffraction demonstrates that the O–H and O···H distances become indistinguishable at 200 K corresponding to the delocalized system (c) [61]. DFT calculations by Deringer et al. [24] demonstrated an agreement with the neutron diffraction data, however, detailed structural data were not provided.

## 3. Hydrogen Atomic Positions of Intramolecular O–H···O Hydrogen Bonds with the Combined use of ^1^H-NMR Chemical Shifts and Quantum Chemical Methods

### 3.1. Factors Influencing OH ^1^H-NMR Chemical Shifts

**Figure 4:**
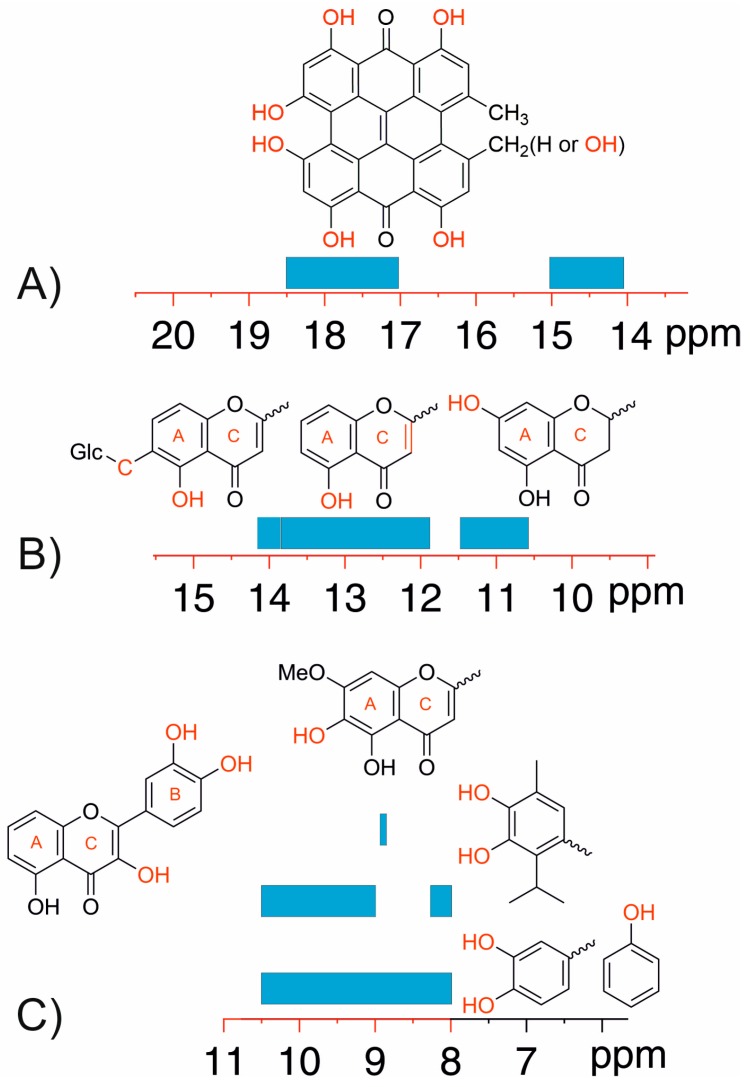
Classification of the chemical shifts of the –OH groups in DMSO-*d*_6_ on the basis of the structure of the molecule and the nature of the substituent in the rings. (**A**–**C**) are the regions of 14–20 ppm, 10–15 ppm, and 8–10.5 ppm, respectively. Reprinted, with permission, from [67]. Copyright 2014, by MDPI.

O–H···O inter- and intramolecular hydrogen bonds have been extensively investigated in the structural analysis of carbohydrates [62,63], natural products [64,65,66,67] (Figure 4), proteins [30,31,68,69] and various hydrogen bonded anions A···H···^−^X of phenols and carboxylic/inorganic acids, (HX) [70,71,72]. Therefore, a large set of experimental data can be used to evaluate the quality of DFT prediction of O–H···O and O–H···^−^O ^1^H-NMR chemical shifts, and the effects of hydrogen bonding interactions and conformation of substituents.

**Figure 5:**
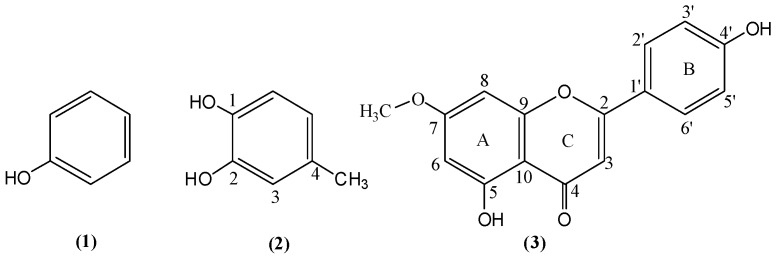
Chemical formulas of phenol (**1**), 4-methylcatechol (**2**), and genkwanin (**3**).

**Figure 6:**
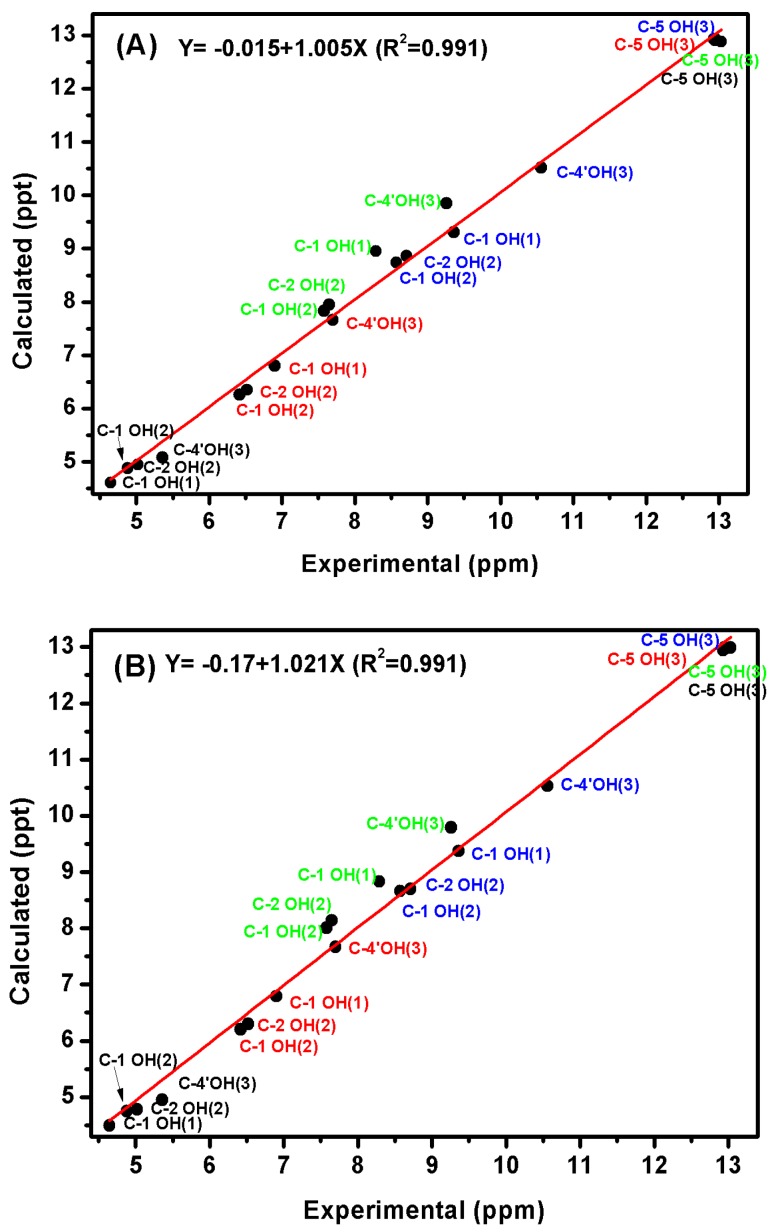
Calculated (at the GIAO DFT/B3LYP/6-311++G(2d,p) level of theory) vs. experimental values of the chemical shifts of the –OH protons of phenol, 4-methylcatehol and genkwanin (Figure 5) in DMSO (yellow), acetone (green), CH_3_CN (red), and CHCl_3_ (blue), with minimization of the complexes with a single solvent molecule at the DFT/B3LYP/6-31+G(d) (**A**) and DFT/B3LYP/6-311++G(d,p); (**B**) level of theory. Adopted, with permission, from [46]. Copyright 2013, The Royal Society of Chemistry.

**Figure 7:**
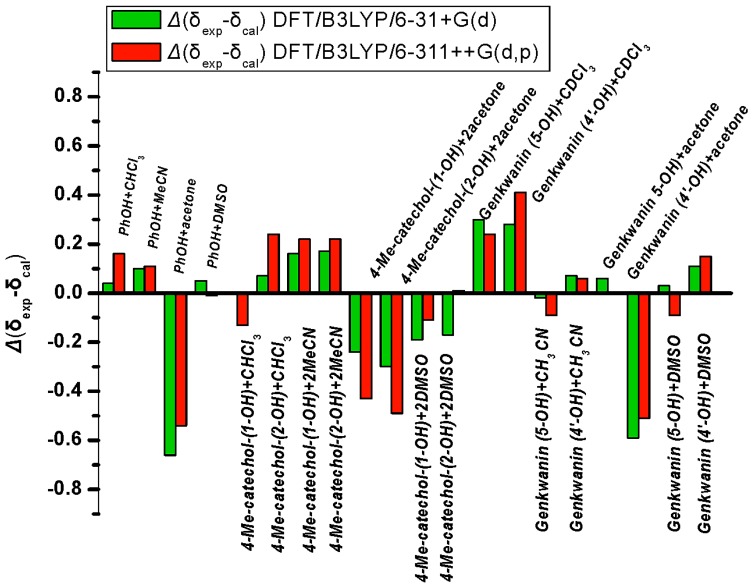
Histogram of the errors Δ(δ_exp_ − δ_cal_) of the calculated (at the GIAO DFT/B3LYP/6-311++G(2d,p) level of theory) ^1^H OH chemical shifts with minimization of the complexes with a single solvent molecule at the DFT/B3LYP/6-31+G(d) and DFT/B3LYP/6-311++G(d,p) level of theory. Reprinted, with permission, from [46]. Copyright 2013, The Royal Society of Chemistry.

Siskos et al. [46] reported that accurate solvent dependent ^1^H-NMR chemical shifts of the –OH groups of phenol (1), 4-methylcatechol (2) and genkwanin (3) (Figure 5) can be obtained, compared to the experimental values, using a combination of DFT, conductor-like polarizable continuum model (CPCM) and discrete solute-solvent hydrogen bond interactions involving a single solvent molecule. In contrast, the calculated ^1^H chemical shifts of phenolic OH groups with the CPCM model, without incorporation of a single solvent molecule, were found to deviate significantly from the experimental value especially in the case of hydrogen bonding solvents. Thus, in DMSO-*d*_6_ the experimental value of the OH group of phenol (1) was found to be 9.36 ppm, while the computed value at 4.27 ppm [46]. Figure 6 illustrates excellent correlation between experimental and calculated (with the GIAO method at the B3LYP/6-311++G(2d,p) level of theory) ^1^H-NMR chemical shifts of the compounds of Figure 5 with minimization of the complexes, with a single solvent molecule, at the B3LYP/6-31+G(d) and B3LYP/6-311++G(d,p) level of theory. The R^2^ values of 0.991 in both cases demonstrate excellent correlation between solvent dependent experimental and calculated ^1^H chemical shifts of phenol OH groups. Furthermore, it was concluded that very large basis sets are not necessary for energy optimization in order to reproduce accurately experimental ^1^H chemical shifts. The histogram of Figure 7 shows that the calculated ^1^H chemical shifts in acetone had the larger error (≥0.3 ppm) with the exception of the C–5 OH of genkwanin. This was attributed to the fact that the plane of the acetone molecule, contrary to the case of DMSO and acetonitrile, deviates from the aromatic plane. The dihedral angle between the planes of the two molecules was found to be strongly dependent on the basis set used [46].

**Figure 8:**
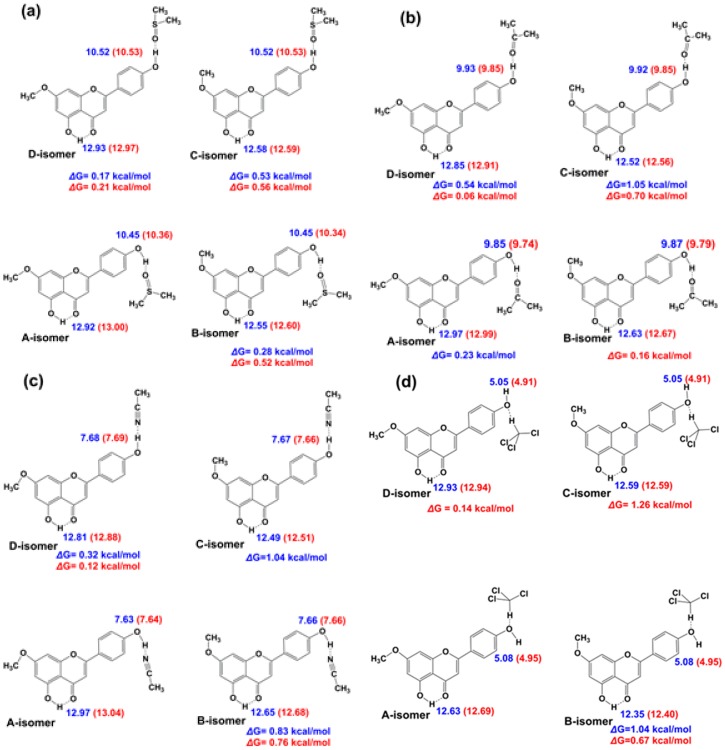
The effect of conformation of the C-7 OCH_3_ and C-4′ OH groups on the chemical shifts of the C-4′ OH and C-5 OH protons for the 1:1 complexes of (**a**) genkwanin + DMSO; (**b**) genkwanin + Me_2_CO; (**c**) genkwanin + MeCN, and (**d**) genkwanin + CHCl_3_, with the use of DFT/B3LYP/6-31+G(d) (data in blue) and DFT/B3LYP/6-311++G(d,p) (data in red), level of theory. Adopted, with permission, from [46]. Copyright 2013, The Royal Society of Chemistry.

A strong intramolecular C–5 OH···OC–4 hydrogen bond was observed in the conformers A to D of genkwanin (Figure 8) with (O)H···O C–4 distance of 1.7056 to 1.7153 Å and 1.6904 to 1.7036 Å at the B3LYP/6-31+G(d) and B3LYP/6-311++G(d,p) level of theory, respectively. An attempt to investigate the interaction of the C–5 OH group of genkwanin with a molecule of DMSO was unsuccessful since the solvent molecule was displaced at a distance >4 Å. This result agrees with detailed experimental data which demonstrated very small temperature and solvent dependence of the C–5 OH ^1^H-NMR chemical shifts in a large number of polyphenolic flavonoid compounds [66]. Figure 8 illustrates the significant effect of conformation of the C–7 OCH_3_ group on the C–5 OH chemical shift of genkwanin. Several conclusions can be drawn from this work [46]: (i) excellent linear correlation between experimental and computed ^1^H-NMR chemical shifts can be obtained without using very large basis sets; (ii) accurate structural and electronic description of solute-solvent interactions can be obtained at a molecular level; (iii) the C–5OH···OC–4 intramolecular hydrogen bond can provide an internal sensor of the conformation of the substituents in ring A of flavonoids (Figure 5).

**Figure 9:**
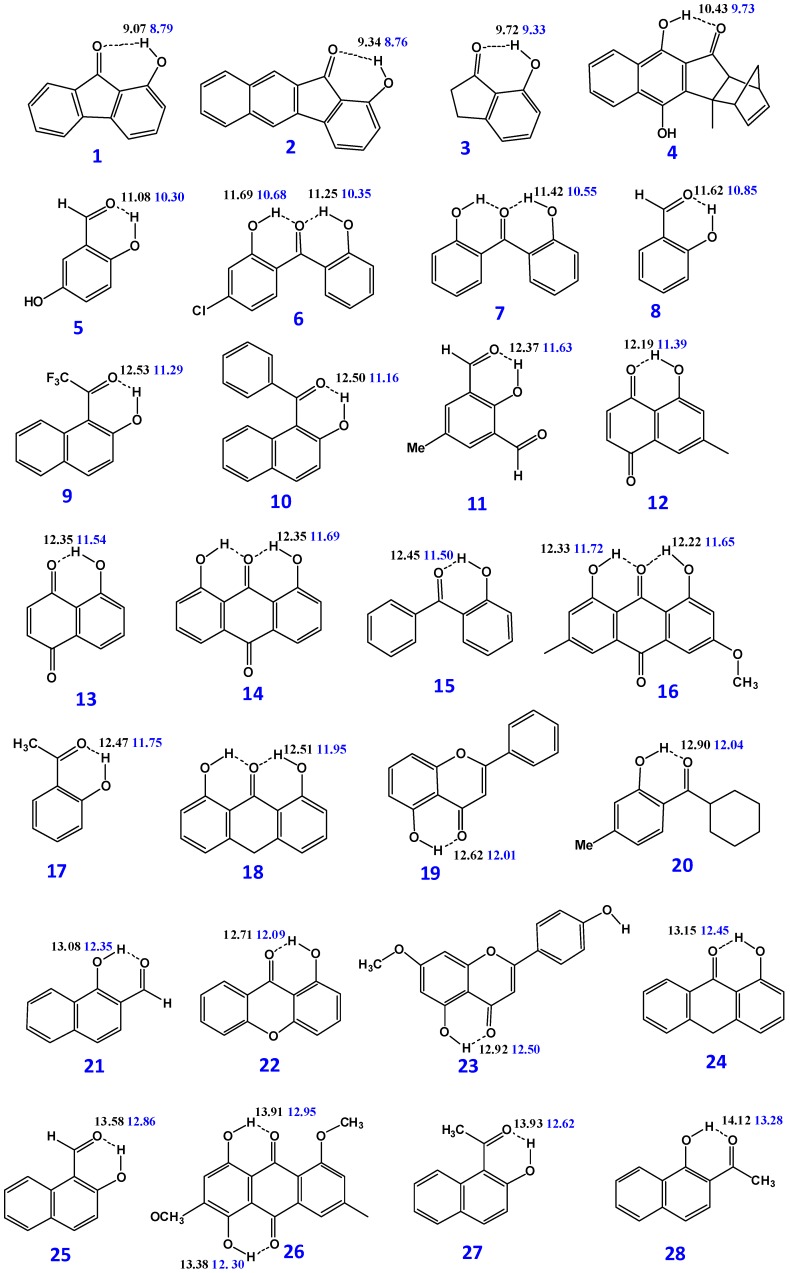

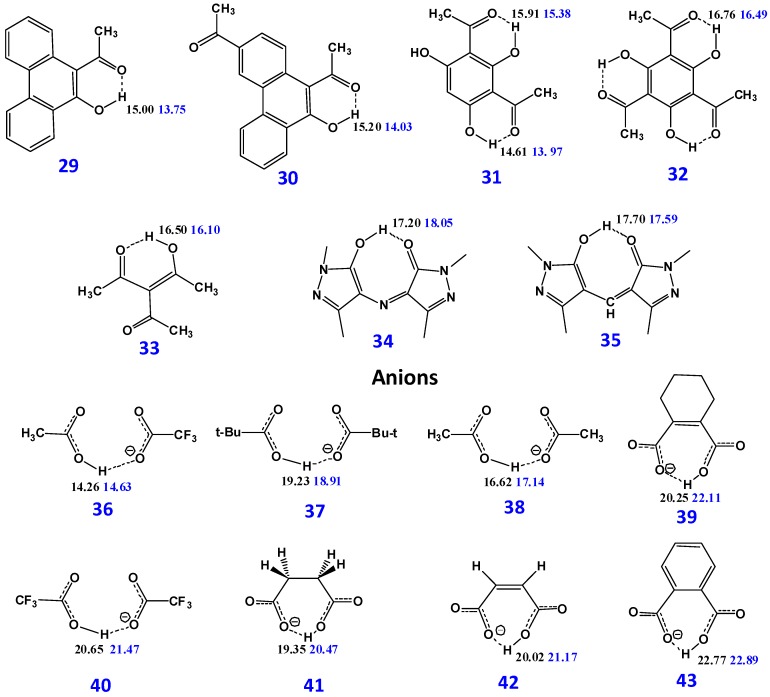
Chemical formulas of phenol compounds exhibiting intramolecular O–H···O hydrogen bonds and ionic complexes with intramolecular and intermolecular O–H···^−^O hydrogen bonds. The data in black and blue are the computed ^1^H chemical shifts, ppm, with minimization of the structures at the B3LYP/6-31+G(d) and M06-2X/6-31+G(d) level of theory, respectively (see Table 1). Reprinted, with permission, from [47]. Copyright 2015, The Royal Society of Chemistry.

**Figure 10:**
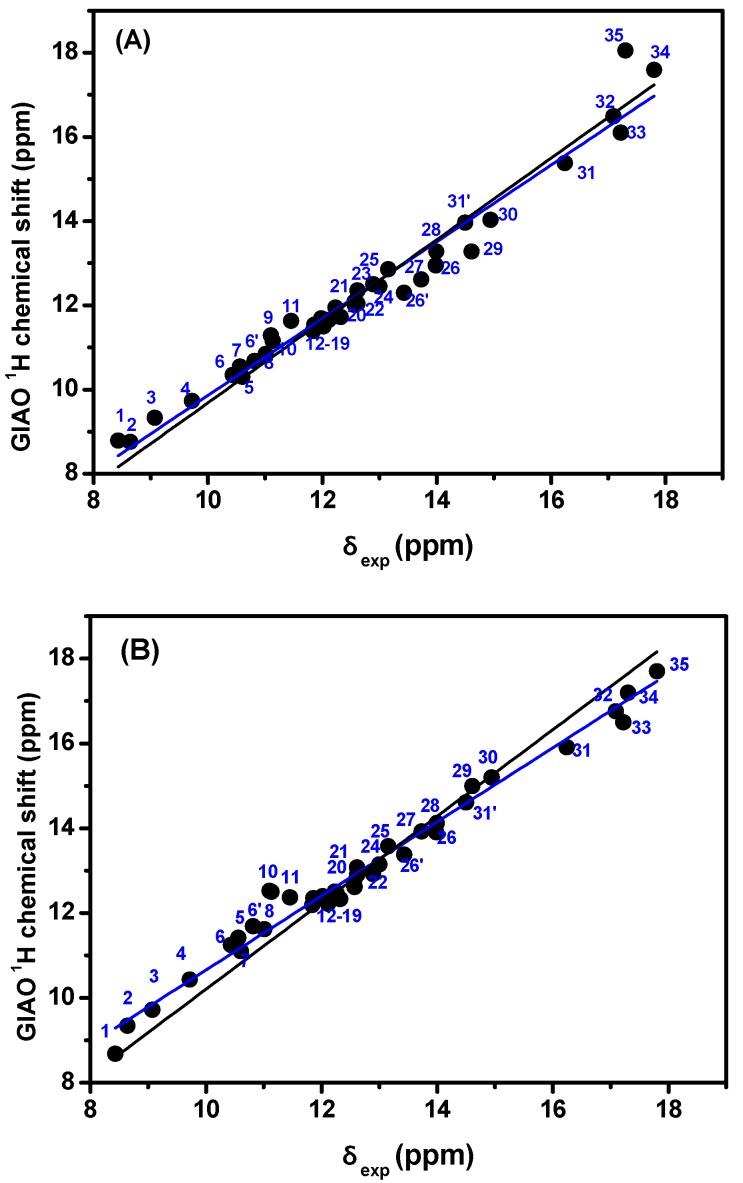
Calculated (at the GIAO/B3LYP/6-311G+(2d,p) level of theory with CPCM in CHCl_3_) vs. experimental chemical shifts of the OH protons of the compounds 1–35 of Figure 9 with minimization of the structures at the M06-2X/6-31+G(d) (**A**) and B3LYP/6-31+G(d) (**B**) level of theory, respectively. The blue line corresponds to the linear fit and the black line to the linear fit through the zero. Reprinted, with permission, from [47]. Copyright 2015, The Royal Society of Chemistry.

Siskos et al. [47] reported an approach for predicting hydrogen bond distances beyond the limits of X-ray diffraction methods, based on quantum chemical calculations of O–H···O ^1^H chemical shifts using a combination of DFT and conductor-like polarizable continuum model (CPCM) in CHCl_3_ without incorporating discrete solvent molecules. For a large set of 35 compounds (Figure 9), exhibiting intramolecular O–H···O hydrogen bonds, very good linear correlations between experimental and computed (at the GIAO B3LYP/6-311++G(2d,p) level of theory) ^1^H chemical shifts were observed with minimization of the structures at the B3LYP/6-31+G(d) and M06-2X/6-31+G(d) level of theory, with coefficients of linear regression R^2^ of 0.977 and 0.965, respectively (Table 1, Figure 10). Further geometry optimizations were performed for selected molecules of Figure 9 with the computationally more demanding MP2/6-31+G(d) level of theory which includes electron correlation effects. The structural characteristics and computed ^1^H chemical shifts were in close agreement with those obtained at the B3LYP/6-31+G(d) and M06-2X/6-31+G(d) level of theory.

The calculated ^1^H chemical shifts of Table 1 were not corrected for quantum zero-point vibrational effects (QZPVE) since detailed GIAO DFT calculations of water clusters (H_2_O)*_n_*, *n* = 2 to 16, with minimization of the structures at the MP2/6-311++G** level of theory, demonstrated that QZPVE effects do not influence ^1^H chemical shifts significantly and, thus, can be neglected [73]. Furthermore, the temperature dependence of ^1^H chemical shifts of phenol OH groups which participate in intramolecular O–H···O hydrogen bonds, exhibit very small Δδ/ΔΤ < 3 ppb in a variety of organic solvents [66] and thus, can be neglected.

Of particular interest are the ionic inter- and intramolecular hydrogen bonded structures 36–43 (Table 1).

**Figure 11:**
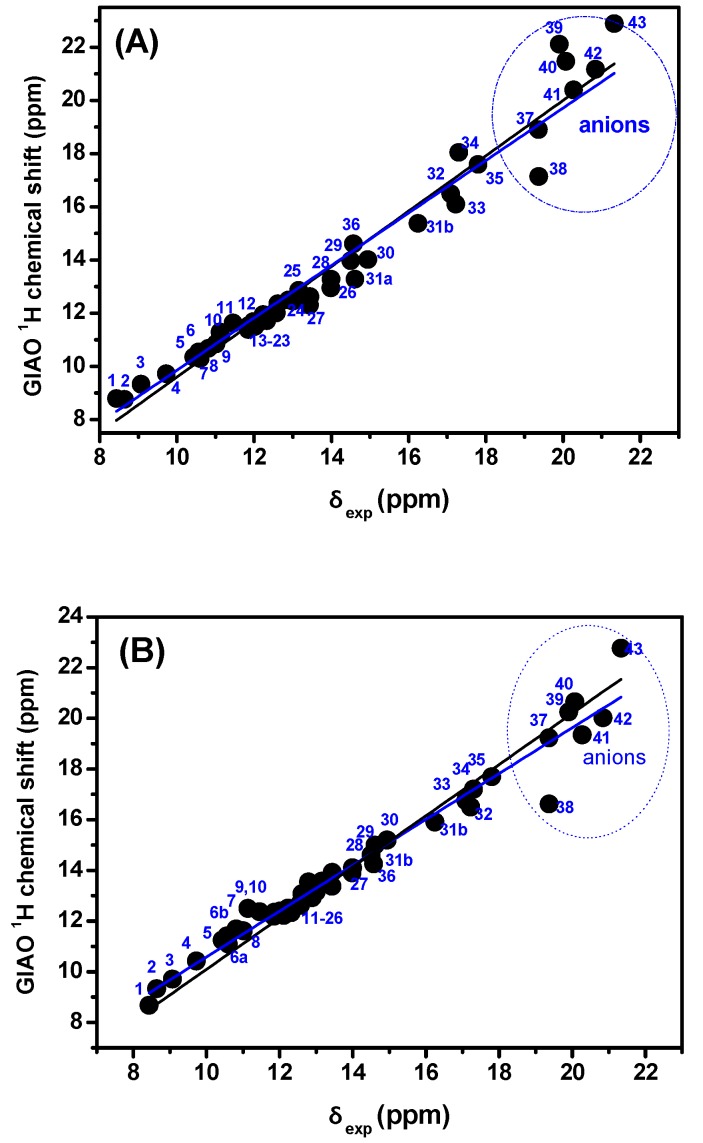
Calculated (at the GIAO/B3LYP/6-311G+(2d,p) level of theory with CPCM in CHCl_3_) vs. experimental chemical shifts of the OH protons of the compounds 1–43 of Figure 9 with minimization of the structures at the M06-2X/6-31+G(d) (**A**) and B3LYP/6-31+G(d) (**B**) level of theory, respectively. The blue line corresponds to the linear fit and the black line to the linear fit through the zero. Reprinted, with permission, from [47]. Copyright 2015, The Royal Society of Chemistry.

Compounds 39, 41, 42 and 43, which exhibit ionic intramolecular hydrogen bonds, adopt a unique low energy conformation. The dimeric complex 36 adopts two low energy conformations: a linear one and a bent structure which is more stable by ~1.43 and 2.73 Kcal·mol^−1^ with the B3LYP/6-31+G(d) and M06-2X/6-31+G(d) basis set, respectively. Similarly, the dimeric complex 38 adopts two low energy conformations with the bent one to be more stable [47]. The dimeric complex 40 adopts a unique linear conformation since the bent structure is of high energy due to repulsive interaction between the –CF_3_ and –COO^−^ groups. The relative stabilities of the linear and bent structures of the dimeric complex 37 (with a *t*-Bu substitution) were found to be strongly dependent on the basis set used. Inclusion in the analysis of the ionic inter- and intramolecular hydrogen bonded structures 36 to 43, results also in very good linear correlation between experimental and computed ^1^H chemical shifts with R^2^ of 0.966 and 0.960 with optimization of the structures at the M06-2X/6-31+G(d) and B3LYP/6-31+G(d) level of theory, respectively (Table 1, Figure 11).

Pylaeva et al. [74] investigated, with the combined use of NMR/UV and ab initio molecular dynamics (MD) proton tautomerism with short ionic O–H···^−^O hydrogen bonds in a complex of 4-nitrophenol with tetraalkylammonium acetate in CD_2_Cl_2_. Ab initio MD with 70 and 71 solvent molecules, with and without the presence of a counter-cation, respectively, demonstrated that the relative motion of the carbonyl group of the acid and the counter-cation plays a major role in the O–H···^−^O bond geometry and in the interconversion of OH···^−^OAc and O^−^···HOAc tautomers. Quantum chemical calculations of the ^1^H-NMR chemical shifts of the bridging proton were performed, at the PBEO/IGLO-III level of theory [75], for 70 random snapshots extracted from the trajectory. A wide range of computational ^1^H chemical shifts were obtained (ca. 14–23 ppm) which reflects an ensemble of structures interconverting in solution. The average value of 18.3 ppm, however, was found to be in reasonable agreement with the experimental value of 17.5 ppm [74].

**Figure 12:**
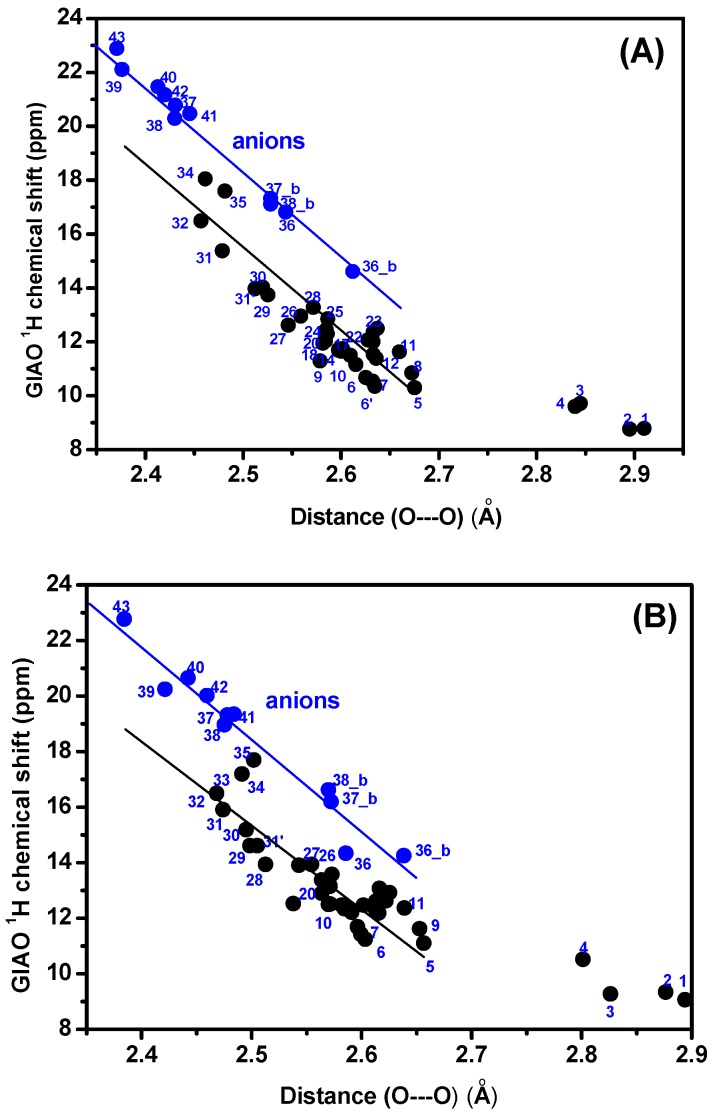
Calculated ^1^H chemical shifts [at the GIAO DFT/B3LYP/6-311+G(2d,p) level of theory with CPCM (CHCl_3_)] vs. O···O distances of the compounds of Figure 9 with minimization of the structures at the M06-2X/6-31+G(d) (**A**) and B3LYP/6-31+G(d) (**B**) level of theory. The notation b refers to the bent complex (see text and Table 1). Reprinted, with permission, from [47]. Copyright 2015, The Royal Society of Chemistry.

Correlations between isotropic chemical shifts and O(H)···O and O···O distances (in parenthesis is the atom which is not taken into consideration in hydrogen bond distances) in O–H···O hydrogen bonds have been established for a variety of organic and inorganic solids [26,28,29,73,76,77,78]. These correlations may be used to obtain the positions of hydrogen atoms in unknown hydrogen bond molecular structures. However, as pointed out by Harris et al. [49] such procedures require prior knowledge of the structures of a series of related compounds. Furthermore, the considerable scatter in several of the plots results in substantial errors in the positions of the hydrogen atoms. Significantly more accurate results can be obtained with the use of quantum chemical calculations of ^1^H-NMR chemical shifts. Figure 12 illustrates a poor correlation between the computed OH ^1^H chemical shift as a function of the computed O···O distances, presumably due to the fact that the oxygen atoms can be thrust together due to steric constraints and, thus, may not be sufficient indicators of hydrogen bond strength [79]. In contrast, a very good linear correlation has been obtained between computed δ(ΟΗ) vs. computed (O)H)···O hydrogen bond distances of the form

δ_ΟΗ_(ppm) = −19.83 r_(O)H...O_+ 46.49 (R^2^ = 0.986) (1)

**Figure 13:**
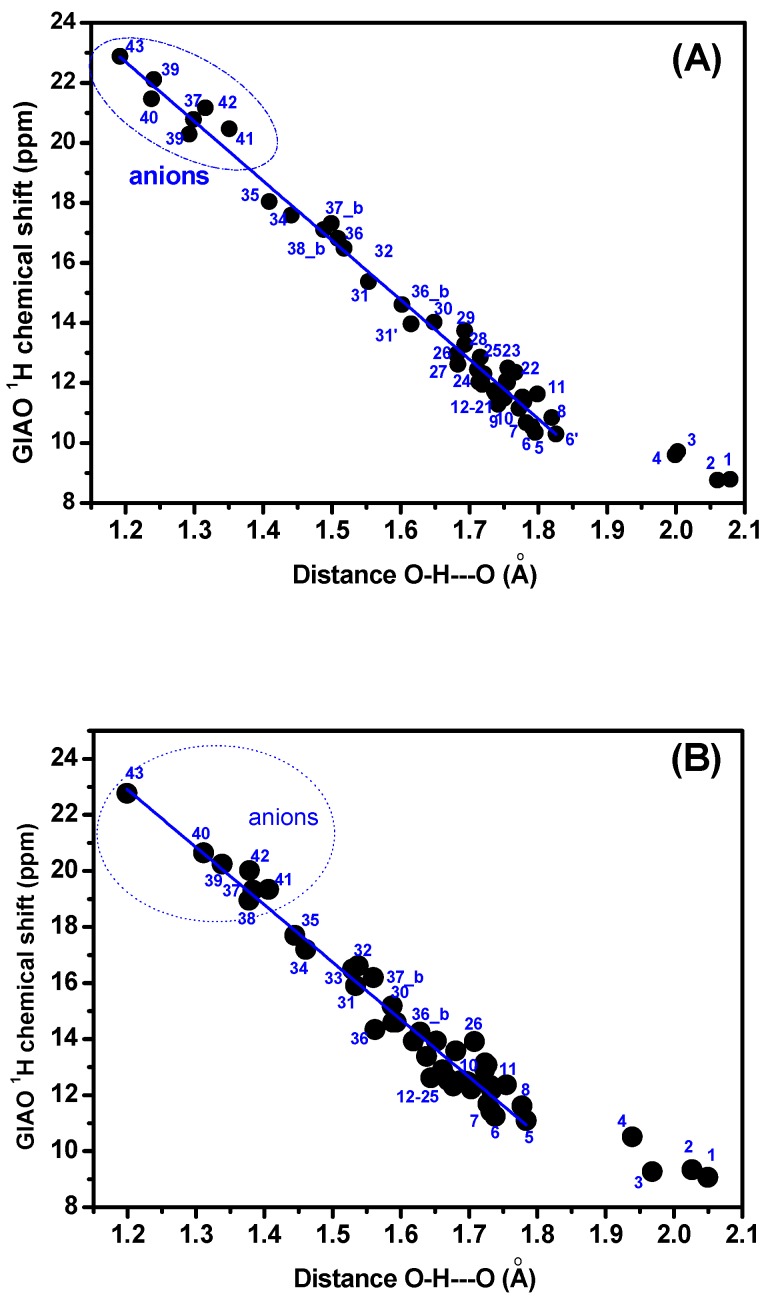
Calculated (at the GIAO/B3LYP/6-311+ G(2d,p) level of theory with CPCM in CHCl_3_) OH proton chemical shifts vs. calculated (O)H···O distances, Å, of the compounds of Figure 9 with minimization of the structures at the M06-2X/6-31+G(d) (**A**) and B3LYP/6-31+ G(d) (**B**) level of theory. The notation b refers to the bent complex (see text and Table 1). Reprinted, with permission, from [47]. Copyright 2015, The Royal Society of Chemistry.

δ_ΟΗ_(ppm) = −20.49 r_(O)H...O_ + 47.49 (R^2^ = 0.961) (2)

for minimization of the structures at the M06-2X/6-31+G(d) and B3LYP/6-31+G(d) level of theory, respectively (Figure 13). The resulting slope of −19.83 Å^−1^ and −20.49 Å^−1^ clearly demonstrate the great sensitivity of δ(OH) upon the (O)H)···O hydrogen bond length. Taking into consideration the statistics of Equations (1) and (2), it was concluded that hydrogen bond distances with an accuracy of ±0.02 Å to ±0.03 Å can be estimated for (O)H)···O and (O)H)···^−^O hydrogen bonds in the range of 1.24 Å to 1.85 Å. Furthermore, it was claimed that quantum chemical calculations of ^1^H chemical shifts can provide hydrogen bond distances of labile hydrogens beyond the limits of X-ray crystallography [47]. Compounds 1–4 were not included in the linear regression analysis since they exhibit relatively weak hydrogen bond with $r_{\left(O\right)H\cdots O}$ > 1.9 Å. Similarly, non-linear behavior has been observed in quantum chemical calculations of acetone-phenol (1:1) complexes at $r_{\left(O\right)H\cdots O}$ > 2.1 Å [80].

**Figure 14:**
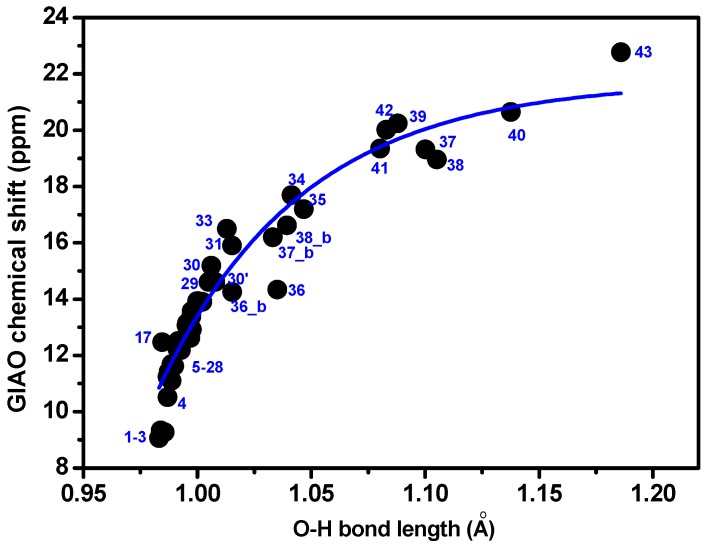
Calculated, at the GIAO/B3LYP/6-311+G(2d,p) level of theory with CPCM in CHCl_3,_ OH proton chemical shifts vs. calculated elongation of the O–H bond, Å, of the structures of the compounds of Figure 9 optimized at the M06-2X/6-31+G(d) level of theory. The notation b refers to the bent complex (see text and Table 1). Adopted, with permission, from [47]. Copyright 2015, The Royal Society of Chemistry.

Figure 14 illustrates a non-linear dependence of the elongation of the O–H bond vs. calculated OH ^1^H chemical shifts at the GIAO/B3LYP/6-311+G(2d,p) level of theory with CPCM in CHCl_3_. The maximum elongation is at ~1.24 Å which corresponds to computed chemical shifts of 21.96 ppm and 21.75 ppm for the optimization of the structures at the B3LYP/6-31+G(d) and M06-2X/6-31+G(d) level of theory, respectively. Interestingly, these values are similar to the experimental value of hydrogen maleate (δ = 20.82 ppm) which exhibits one of the strongest symmetric hydrogen bonds [81,82].

Table 1 represents the natural bond orbital (NBO) charges of the atoms participating in O–H)···O and O–H)···^−^O intra- and intermolecular hydrogen bonds, calculated at the B3LYP/6-31+G(d) and M06-2X/6-31+G(d) level of theory. The NBO charge of the hydrogen atom indicates an insignificant variation over the whole range of the compounds 1–43. The charge of the proton acceptor oxygen increases from −0.578 for 1 up to −0.772 for the compound 43 at the M06-2X/6-31+G(d) level of theory. The charge of the proton donor oxygen shows a moderate increase from −0.706 for 1 up to 0.770 for compound **43** at the M06-2X/6-31+G(d) level of theory. Similar results were obtained at the B3LYP/6-31+G(d) level of theory. The difference in the magnitude of the NBO charges, ΔQ × 10^3^, of the two oxygens indicates no functional correlation with ^1^H chemical shifts (Table 1) [47].

Mori and Masuda [83] investigated the effect of solvent on proton location and dynamic behavior in dibenzoylmethane (Scheme 1) with the use of molecular dynamics simulations and NMR experiments. The potential energy surface of dibenzoylmethane is considered to be a double minimum due to tautomeric equilibrium of the two forms (a) and (b) (Scheme 1) with a low barrier, based on the experimental large positive H/D isotope effect [84]. DFT calculations of the enol form in vacuum indicated that the barrier height for the intramolecular proton transfer is 5.4 KJ·mol^−1^ and very similar to the zero-point energy of the vibrational ground state of 5.1 KJ·mol^−1^, resulting in broad distribution of the proton density along the hydrogen bond. Specific interactions with protic solvents or strong hydrogen bond accepting solvents affect the geometry and dynamic behavior of the intramolecular hydrogen bond of dibenzoylmethane. The averaged hydrogen bond geometries sampled by the path integral MD simulations were found to be in much better agreement with the experimental results than those obtained by the classical MD simulations [83].

There are numerous experimental and theoretical studies on the importance of covalency in hydrogen bonding phenomena [85,86,87]. If the O–H bond participates in a hydrogen bond interaction, then, the O–H bond elongates and consequently the bond number value decreases. This loss is compensated by the H···O interaction due to sharing of the lone pair of electrons of the accepting oxygen atom. A fractional part of the electron pair is expressed by the bond order. Hence, short O···O contacts within the O–H···O systems, result in longer O–H bonds. There are numerous neutron diffraction results on O–H···O systems and several studies concerning the relationships between the above geometrical parameters [88].

**Figure 15:**
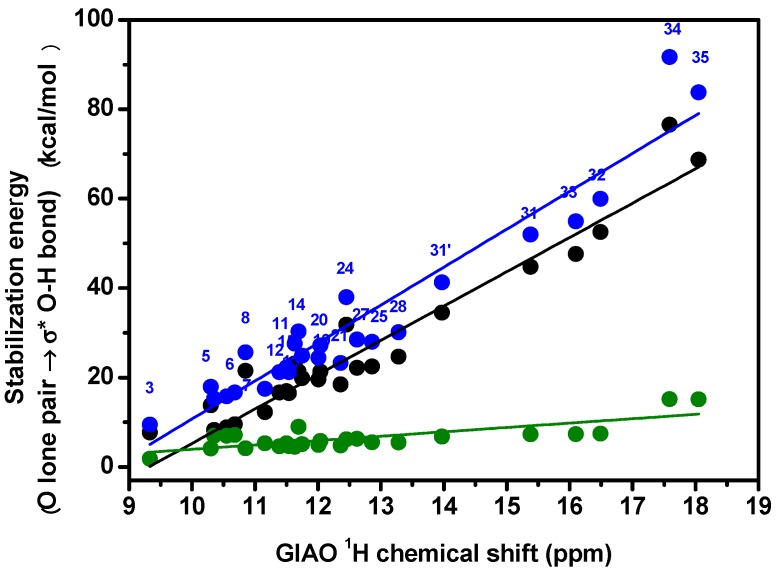
Calculated, at the GIAO DFT/B3LYP/6-311+G(2d,p) level of theory with CPCM in CHCl_3_, chemical shifts of the compounds of Figure 9 with minimization of structures at the M06-2X/6-31+G(d) level of theory) vs. stabilization energy (Second Order Perturbation Theory Analysis) between the lone pairs of the oxygen of the carbonyl group with the σ* antibonding orbital of the H-O bond) (lone pair 1 in black, R^2^ = 0.946, lone pair 2 in green, R^2^ = 0.575, and the sum in blue, R^2^ = 0.921). Reprinted, with permission, from [47]. Copyright 2015, The Royal Society of Chemistry.

**Figure 16:**
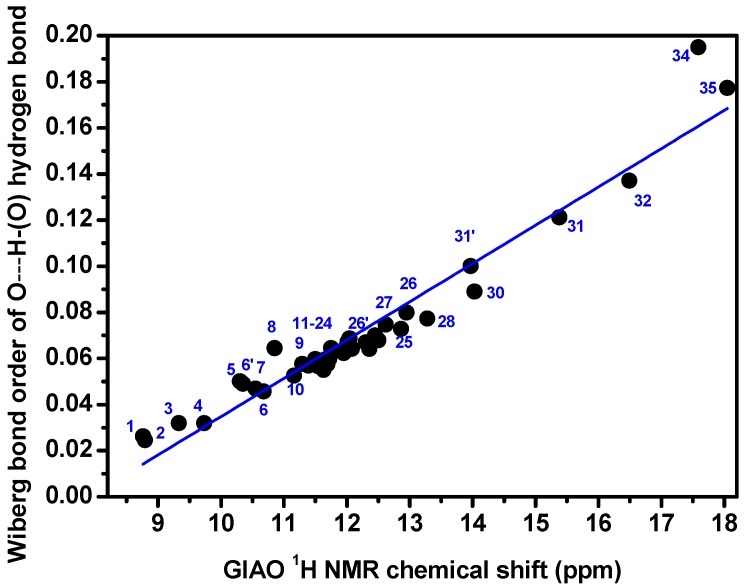
Plot of calculated Wiberg bond order of the intramolecular O···H(O) hydrogen bond of the compounds 1–35 of Scheme 3 vs. GIAO calculated ^1^H chemical shifts. The minimization of the structures and the NBO analysis were performed at the M06-2X/6-31+G(d) level of theory. Adopted, with permission, from [47]. Copyright 2015, The Royal Society of Chemistry.

The hydrogen bond formation in O–H···O systems may be considered as a combination of two electronic processes. A charge transfer from the oxygen lone pair to the σ*(O–H) antibonding orbital and an increase in s-character of the oxygen hybrid orbital. The first process leads to the elongation of the O–H bond and shortening of the O–H···O hydrogen bond, while the increase in s-character results in the reverse effect. Figure 15 shows a very good correlation (R^2^ = 0.946) between the calculated ^1^H chemical shifts and the second-order stabilization energies, $E_{LP\left(c\right)\to \sigma_{ΟΗ}^{*}}^{\left(2\right)}$ of the charge transfer between the oxygen lone pair and $\sigma_{ΟΗ}^{*}$ antibonding orbital. Similarly, a very good linear correlation between GIAO δ(^1^Η) and Wiberg bond order of the intramolecular O–H···O was observed (Figure 16). On the contrary, GIAO δ(^1^H) shows no functional relationship with the charge density of the proton participating in hydrogen bonding. The concept, therefore, of a three center – four electron sharing due to resonance type n_B_ → $\sigma_{ΑΗ}^{*}$ interaction:

O–H: O ↔ O: H–O

may be the basis for a revised IUPAC definition of the hydrogen bond [89]. This linear correlation between Wiberg bond order and δ(^1^H) may resolve the ambiguity that a deshielded bridging proton signal is a necessary but not sufficient demonstration of a hydrogen bond [90]. As pointed out by Scheiner [90,91], deshielding effects can be observed in cases of the proximity of an electron cloud of a second molecule even if no hydrogen bond is present.

### 3.2. Resolving Conflicting Literature OH Resonance Assignments

Computational modeling of ^1^H and ^13^C chemical shifts has been widely used to assist in the assignment or reassignment of experimental spectra and in the elucidation of chemical structures [45]. Chemical shifts of hydroxyl groups, however, have rarely been investigated at the quantum chemical level. This may be attributed to the fact that –OH groups displayed broad signals and variable chemical shifts at room temperature due to intermolecular exchange of the –OH protons with protons of the protic solvents or with protons of the residual H_2_O in aprotic solvents. However, optimization of experimental conditions, such as pH, temperature and NMR solvent may result in obtaining extremely sharp peaks (Δν_1/2_ ≤ 2 Hz) which can lead to unequivocal assignment of the OH signals [64,65,66,67,92,93,94].

**Figure 17:**
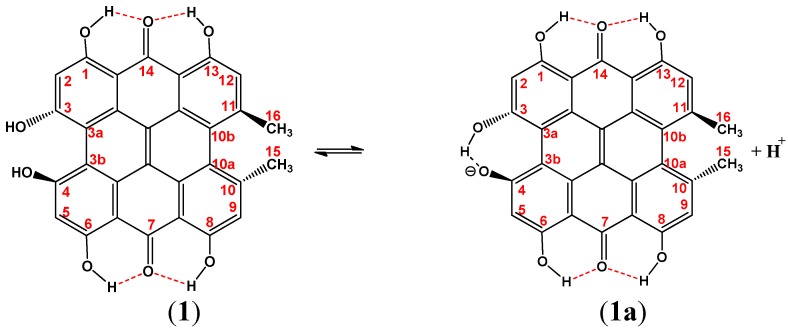
Solvent dependent equilibrium of the 7,14-dioxo tautomer of hypericin HyH, Q^7,14^ (**1**), and its anionic form Hy^−^ (**1a**).

**Figure 18:**
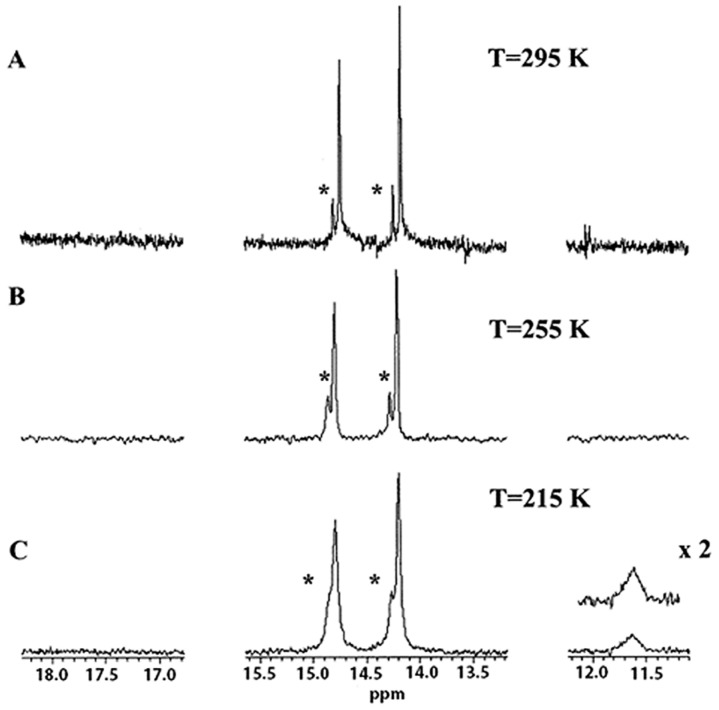
Variable temperature gradient ^1^H-NMR spectra of hypericin in acetone-*d*_6_ (neutral form (**1**), Figure 17), number of scans 128, 754 and 1952 for A, B and C, respectively. The asterisk denotes an unknown compound. Adopted, with permission, from [101]. Copyright 2002, by Elsevier Science Ltd.

**Figure 19:**
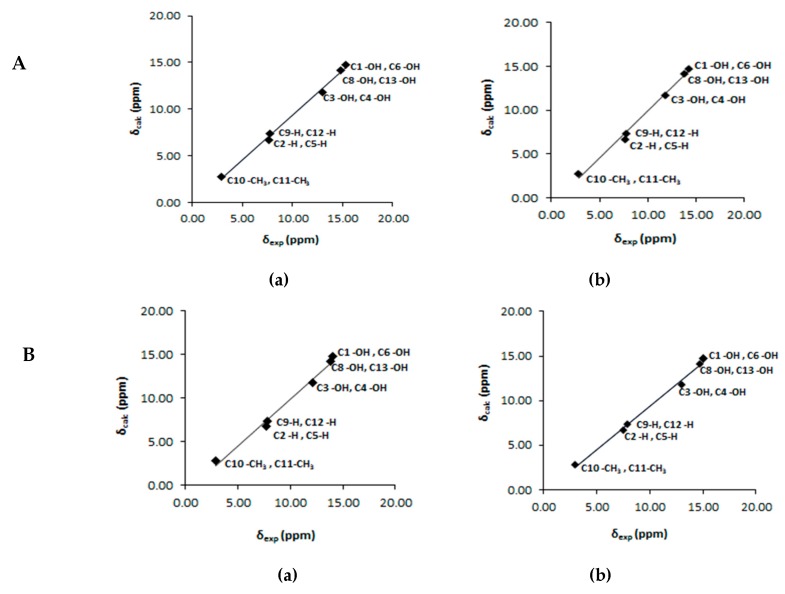
(**A**) Calculated (δ_calc_, ppm) (at the GIAO B3LYP/6-311+G(2d,p) level of theory) vs. experimental values (δ_exp_, ppm) of the ^1^H-NMR chemical shifts of neutral hypericin + 1 molecule of acetone with minimization of the structures at the B3LYP/6-31+G(d) (CPCM) (**a**) and at the TPSSh/TZVP (CPCM) (**b**), level of theory, respectively; (**B**) Calculated (δ_calc_, ppm) (at the GIAO B3LYP/6-311+G(2d,p) level of theory) vs. experimental values (δ_exp_, ppm) of the ^1^H-NMR chemical shifts of neutral hypericin + 2 molecules of acetone with minimization of the structures at the B3LYP/6-31+G(d) (CPCM) (**a**) and at the TPSSh/TZVP (CPCM) (**b**) level of theory, respectively. Reprinted, with permission, from [50]. Copyright 2016, by Elsevier Science Ltd

Hypericin (phenanthro[1,10,9,8,o,p,q,r,a]perylane-7,14 dione]), Figure 17, is one of the principal active phytochemicals of Hypericium (Saint John’s wort) [95,96]. It is very acidic with p*K*_a_ = 1.7–2.0 due to the hydroxyl group on C-4(C-3) [97,98]. ^1^H-NMR and 2D NOESY spectroscopy in DMSO-*d*_6_ and acetone-*d*_6_ indicated that hypericin exists in both solvents in the neutral form (Figure 17(1)) and the bay O–H 3,4 protons appear at ~8.1–8.3 ppm [99]. Dax et al. [100] suggested that hypericin in DMSO-*d*_6_ exists in the ionic form on the basis of a strong deshielded resonance at δ = 17.3–17.5 ppm. Skalkos et al. [101] with the use of variable temperature ^1^H-NMR concluded that hypericin in acetone-*d*_6_ exists in the neutral form and the bay O–H 3,4 protons appear as a broad composite signal at ~11.6–11.8 ppm (T = 215 K, Figure 18C). Siskos et al. [50] performed detailed DFT calculations of ^1^H-NMR chemical shifts of hypericin (1) and its ionic form (1a) using various basis sets. Table S1 illustrates calculated ^1^H-NMR chemical shifts of various hypericin + solvent (1:1 and 1:2) complexes with the GIAO/B3LYP/6-311+G(2d,p)-CPCM [102] method. The internuclear distance between the bay C-3 OH hydrogen and the oxygen of the acetone or DMSO solvation molecule was found to be strongly dependent on the basis set used and whether the gas phase or the CPCM model was used. Thus, the respective internuclear distance with the oxygen atom of the single DMSO solvation molecule reduces from 1.625 Å to 1.494 Å at the B3LYP/6-31+G(d) (gas phase) and TPSSh/TZVP (IEF-PCM) level of theory, respectively. This pronounced shortening in hydrogen bond distance, was attributed to the very strong cooperative effect of the intramolecular hydrogen bond of the two bay OH groups which also shows a significant variation as a function of the basis set used (1.630 Å and 1.549 Å for minimization of the structures at the B3LYP/6-31+G(d) (gas phase) and TPSSh/TZVP (IEF-PCM) level of theory, respectively. Figure 19 illustrates plots between experimental and calculated ^1^H chemical shifts of hypericin using the experimental value of δ = 11.75 ppm in acetone-*d*_6_ of the O–H 3,4 bay protons suggested by Skalkos et al. [101]. Excellent linear correlations were obtained with R^2^ ~0.985–0.997. The use of δ = 8.2 ppm for the O–H 3,4 protons suggested by Smirnov et al. [99] results in a reduction in the correlation coefficient (R^2^ ≈ 0.852–0.923) which clearly demonstrates an erroneous assignment (Table S2).

### 3.3. Comparison of X-ray with NMR Crystallography in the Solid State—The Location of Labile Hydrogens

Assignment of ^1^H-NMR resonances in the solid state is very challenging due to strong homonuclear dipolar couplings which, in several cases, exceed a significant range of chemical shifts of protons. However, in cases of hydrogen bonding interactions the strong deshielding may result in well resolved resonances which can be assigned unambiguously. Harris et al. [49] reported that the chemical shift of the proton in an NH···O=C hydrogen bond is primary dependent on the distance between the proton and the donor and showed that a combined experimental high speed MAS ^1^H-NMR and DFT calculations of ^1^H chemical shifts can be used to refine the hydrogen position of X-ray structures. It was suggested that the method provides better results than either X-ray diffraction or NMR dipolar couplings [41,42].

**Figure 20:**
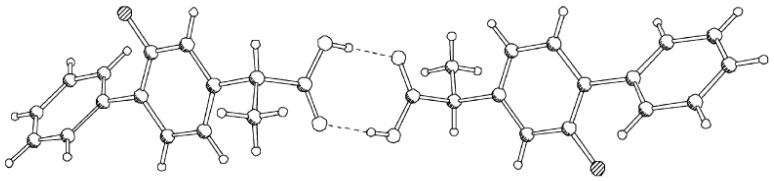
Hydrogen bonded dimer of flurbiprofen (Form 1) based on the published X-ray structure [106], but with adjustment of the hydrogen atoms as obtained by computation. Reprinted, with permission, from [105]. Copyright 2005, The Royal Society of Chemistry.

**Figure 21:**
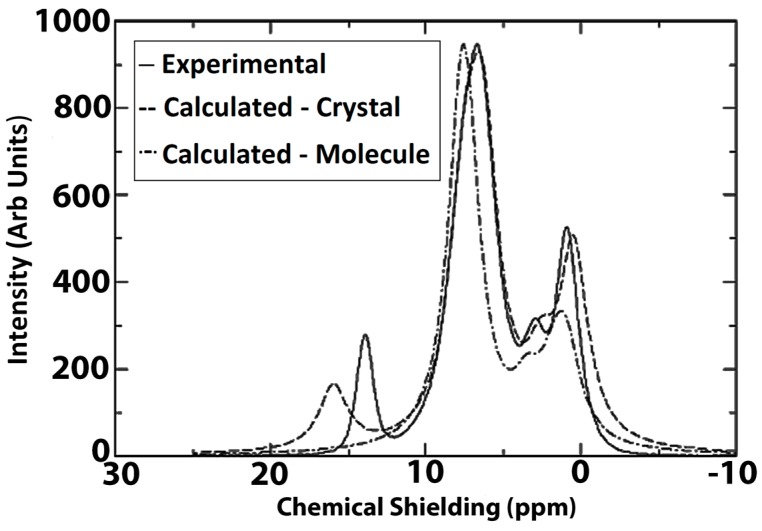
Experimental and simulated, using geometries with only the proton positions relaxed, ^1^H-NMR spectrum of flurbiprofen in the solid state. Reprinted, with permission, from [105]. Copyright 2005, The Royal Society of Chemistry.

The application of quantum chemical methods or solid materials was greatly improved with the development of the gauge including projector augmented wave (GIPAW) approach, which enabled the calculation of magnetic shielding of an inherently periodic solid using a plane wave basis set [103,104]. Thus, accurate calculations of all atoms within a small volume unit can be extended in a repeating three-dimensional structure with a significant saving in computer time. Yates et al. [105] reported calculations of ^1^H, ^13^C and ^19^F shielding parameters of flurbiprofen, a non-steroidal anti-inflammatory drug, under periodic boundary conditions in the crystalline lattice using DFT with the GIPAW method. The compound contains hydrogen bonds between the carboxylic functions of two adjacent molecules in the crystal (Figure 20). The O–H bond length of the hydrogen optimized structure is shorter by 0.263 Å than the reported X-ray structure [106] (Table 2) and in agreement with neutron diffraction data of similar compounds [2]. Calculations of the ^1^H shielding within the periodic boundary condition results in chemical shifts of 17.8 and 15.9 ppm for the fully optimized and hydrogen optimized structures, respectively. These values should be compared with the experimental value of ~14 ppm (Figure 21). When the X-ray structure was used to simulate the ^1^H solid state NMR spectrum of flurbiprofen the OH chemical shift was calculated to be ~22.3 ppm, which is significantly different from the experimental value. The authors concluded that ^1^H chemical shifts are extremely useful parameters for the fine adjustment of the hydrogen bond geometry.

More recently, the potential of ^1^H-NMR crystallography was demonstrated in the evaluation of X-ray structures of furosemide which differ only in the displacement of the COOH hydrogen atom [107]. The RMSD values for δ_iso_ (^1^H) between experimentally measured δ_iso_ and those computed using GIPAW DFT on hydrogen optimized structures were shown to be very sensitive to the COOH hydrogen atomic position. The X-ray structure with a hydrogen bonding motive where one of the COOH hydrogen atom is directed away from forming a classical O–H···O dimer has a significantly larger RMSD values for ^1^H and, thus, does not meet the verification criteria.

Baias et al. [108] reported a protocol for the quantum chemical structure determination of powdered solids by combining solid state NMR spectroscopy and DFT chemical shift calculations. The assigned ^1^H isotropic chemical shifts for three drug molecules (cocaine, flutamide and flurfenamic acid, but not for theophylline) were sufficient to determine the correct structures from a set of predicted structures using the root-mean-square deviation between experimentally determined and calculated chemical shifts. The authors did not utilize NH or OH protons in the structural analysis due to the temperature dependence of the chemical shifts. In our opinion, the effect of temperature is much smaller than the effect of hydrogen bonding and, furthermore, a correction can be made based on temperature coefficients of chemical shifts [32,66].

Phillip et al. [109,110] investigated whether NMR crystallography methods can be used to probe complex hydrogen bonding networks in quercetin dihydrate, of known X-ray single crystal structure [111], and in anhydrous quercetin. A computational method was proposed for ranking all possible conformers of the five OH hydroxyl groups of quercetin in the crystalline environment as follows: a quick systematic search in the conformational space was carried out at the molecular mechanics level of theory, whereas for relaxing the initial molecular conformers in the crystalline environment, DFT geometry optimization of hydrogen atomic positions was applied. A very limited ^1^H-NMR spectral resolution, however, was obtained especially in the case of anhydrous quercetin even at ultrafast MAS frequency at 60 KHz and with the use of 2D DQ-SQ correlation. Thus, only the resonance of the strongly deshielded C(5)–OH proton at 12.4 and 13.2 ppm, due to the formation of a strong intramolecular C(5)–OH···O(C-4) hydrogen bond in the two samples, was unequivocally assigned.

### 3.4. Comparison of X-ray with NMR Crystallography in Solution—The Location of Labile Hydrogens

Recently, Siskos et al. [50] investigated the position of labile hydrogen involved in O–H···O intramolecular hydrogen bond in hypericinate (Figure 17). Table 3 illustrates a comparison of intramolecular hydrogen bond distances and dihedral angles of the calculated structures, at the B3LYP/6-31+G(d) (IEF-PCM in DMSO) and at the TPSS/TZVP (IEF-PCM in DMSO) level of theory, of the hypericine ion (1a) with those of the X-ray structure. The calculated intramolecular distances of the heavy atoms, such as the O(1)···O(14), O(6)···O(7), O(3)···O(4), O(13)···O(14) and O(8)···O(7) distances, were found to be in excellent agreement with those of the X-ray structure [97]. Similarly, the dihedral angles C(3)–C(3a)–C(3b)–C(4) and C(10)–C(10a)–C(10b)–C(11) were shown to indicate minor differences. On the contrary, significant differences have been observed in the location of all hydrogen atoms involved in intramolecular hydrogen bonds between the calculated and the X-ray structure, most notably in the O(1)–H(1) and O(13)–H(13) bond distances which deviate by 0.11 Å.

**Figure 22:**
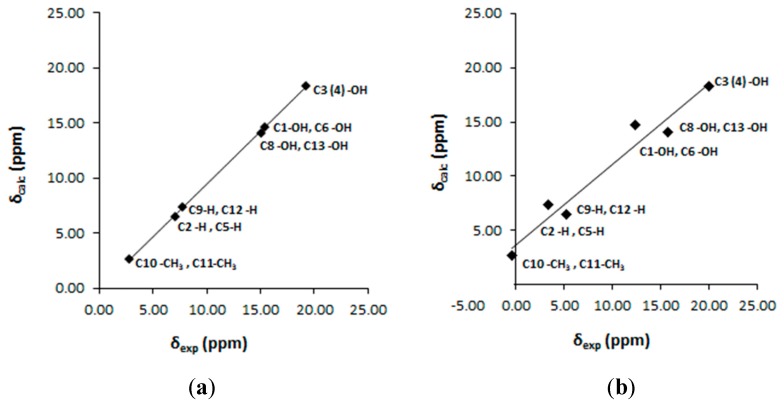
Calculated (δ_calc_) (at the GIAO DFT B3LYP/6-31 +G(2d,p) level of theory) vs. experimental values (δ_exp_) of the ^1^H-NMR chemical shifts of hypericinate with minimization of the structure at the TPSSh/TZVP (IEF-PCM) level of theory, (**a**) and with the use of the X-ray structure [78] as input geometry; (**b**) Reprinted, with permission, from [50]. Copyright 2016, by Elsevier Science Ltd.

Figure 22 illustrates calculated (δ_calc_) at the GIAO DFT B3LYP/6-31+G(2d,p) level of theory, vs. experimental values (δ_exp_) of the ^1^H chemical shifts of hypericinate with minimization of the structure at the TPSSh/TZVP (IEF-PCM) level of theory. The resulting R^2^ value (~0.994), slope (~0.954) and mean square error (~0.027 ppm) clearly demonstrate an excellent correlation. On the contrary, when the X-ray structure was used as an input geometry, the resulting R^2^ value (~0.968), slope (~0.747) and mean square error (~1.443 ppm) indicate a modest correlation (Figure 22b).

## 4. Conclusions

From the reports discussed herein it is evident that the field of NMR crystallography of labile hydrogens is in the early development and that there is a need for further improvement in the efficacy and accuracy of computation. Additional advances may require the automatic structure refinement based on NMR data and the inclusion of temperature effects into calculation for a direct comparison with the experiment [53]. Nevertheless, the recent reports provide substantial evidence that the application of accurate quantum chemical methods can be an excellent means to investigate hydrogen atomic positions in hydrogen bonds. More specific, the linear correlation between DFT calculated and experimental OH ^1^H-NMR chemical shift, even for moderate basis sets, and the great sensitivity of ^1^H chemical shifts to hydrogen bonding properties can provide an excellent method:

(i) in resolving conflicting literature data and ambiguities in resonance assignment;
(ii) in determining accurate labile hydrogen positions beyond the limits of X-ray diffraction methods, NMR dipolar methods [41,42] or spin diffusion [112] and
(iii) in investigating the nature of hydrogen bonding.

Further studies may include DFT calculations of O–H···O (and similarly N–H···O) ^1^H chemical shifts in investigating enol-enol tautomeric equilibrium and comparison with indirect literature experimental methods based on interpolation of ^13^C and ^17^O chemical shifts [59,113,114,115], isotope effects [39,116], *^n^J*(^13^C,O^1^H) coupling constants [117] and in resolving the key controversy on the shape of the O–H···O intramolecular hydrogen bond potential function [9,58,59,60,118].

We hope that this review will aid structural chemistry community in adding quantum chemical methods and especially calculations of ^1^H-NMR chemical shifts to their repertoire of methods for high resolution structures of hydrogen bonding interactions.

## Figures and Tables

**Table 1:** Calculated (δ,ppm) GIAO/DFT/B3LYP/6-311+G(2d,p) (geometry optimization at the B3LYP/6-31+G(d), in black, and M06-2X/6-31+G(d), in blue, level of theory) and experimental (δ_exp_, ppm) ^1^H chemical shifts, (O)H···O and O–H bond distances, natural bond order (NBO) charges of the O–H···O atoms, and NBO charge differences of the two oxygens of the hydrogen bond (ΔQ × 10^3^) of the compounds of Figure 9. Reprinted, with permission, from [47]. Copyright 2015, The Royal Society of Chemistry.

Compounds	B3LYP/6-31 + G(d) δ (ppm)	M06-2X/6-31 + G(d) δ (ppm)	δ_exp_ (ppm)	(O)H···O (Å)	O–H (Å)	NBO O···H-O	ΔQ × 10^3^
**1**	9.07	8.79	8.76	2.04923	0.98315	(−0.578, +0.533, −0.690)	112
				2.07842	0.97824	(−0.572, +0.541, −0.706)	134
**2**	9.34	8.76	8.64	2.02634	0.98385	(−0.588, +0.533, −0.691)	103
				2.05999	0.97875	(−0.579, +0.543, −0.706)	127
**3**	9.72	9.33	9.07, 9.04	1.96789	0.98563	(−0.604, +0.533, −0.693)	89
				2.00225	0.97982	(−0.601, +0.543, −0.709)	108
**4**	10.43	9.73	9.72	1.93876	0.98692	(−0.612, +0.537, −0.695)	83
				1.99843	0.98047	(−0.608, +0.547, −0.711)	103
**5**	11.01(49.65%) 11.15(50.35%) 11.08	10.08 (48.5%) 10.47(51.5%) 10.30	10.6	1.78342	0.98868	(−0.593, +0.533, −0.699)	106
				1.78486	0.98866	(−0.595, +0.533, −0.697)	102
				1.82655	0.97982	(−0.590, +0.544, −0.718)	128
				1.82531	0.98079	(−0.592, +0.545, −0.716)	124
**6**	11.25 11.69	10.35 10.68	10.43 10.81	1.73815	0.98692	(−0.672, +0.536, −0.696)	24
				1.72708	0.98862	(−0.672, +0.538, −0.692)	20
				1.79525	0.97878	(−0.667, +0.547, −0.714)	47
				1.78196	0.98027	(−0.667, +0.549, −0.710)	43
**7**	11.42	10.55	10.54	1.73187	0.98741	(−0.673, +0.536, −0.698)	25
			10.59	1.79045	0.97921	(−0.668, +0.547, −0.716)	48
**8**	11.62	10.85	11.01	1.77701	0.98985	(−0.596, +0.534, −0.693)	97
				1.81916	0.98173	(−0.595, +0.546, −0.712)	117
**9**	12.53	11.29	11.10	1.66898	0.99237	(−0582, +0.536, −0.678)	96
				1.74125	0.98160	(−0.576, +0.548, −0.699)	123
**10**	12.50	11.16	11.13	1.69508	0.99237	(−0.617, +0.537, −0.693)	76
				1.77197	0.98155	(−0.611, +0.548, −0.712)	101
**11**	12.37	11.63	11.45	1.75446	0.99152	(−0.588, +0.534, −0.657)	69
				1.79817	0.98306	(−0.587, +0.549, −0.679)	92
**12**	12.19	11.39	11.84	1.73269	0.99280	(−0.591, +0.536, −0.690)	99
				1.77877	0.98324	(−0.589, +0.548, −0.710)	121
**13**	12.35	11.54	11.86	1.73107	0.99232	(−0.586, +0.536, −0.689)	103
				1.77694	0.98291	(−0.584, +0.548, −0.710)	126
**14**	12.35	11.69	11.98	1.70550	0.99079	(−0.666, +0.538, −0.690)	24
				1.74172	0.98252	(−0.669, +0.549, −0.709)	40
**15**	12.45	11.50	12.02	1.69731	0.99279	(−0.611, +0.535, −0.698)	87
				1.75040	0.98318	(−0.608, +0.548, −0.717)	109
**16**	12.22 12.33	11.65 11.72	12.11 12.32	1.70733	0.98130	(−0.674, +0.538, −0.694)	20
				1.73743	0.98316	(−0.678, +0.549, −0.711)	33
				1.70310	0.99197	(−0.674, +0.538, −0.693)	19
				1.73576	0.98362	(−0.678, +0.549, −0.709)	31
**17**	12.47	11.75	12.26	1.69790	0.99348	(−0.610, +0.534, −0.698)	85
				1.73631	0.98459	(−0.609, +0.547, −0.717)	108
**18**	12.51	11.95	12.23	1.68655	0.99150	(−0.679, +0.536, −0.697)	18
				1.71821	0.98338	(−0.585, +0.548, −0.714)	29
**19**	12.62	12.01	12.57	1.72324	0.99693	(−0.637, +0.536, −0.699)	42
				1.75500	0.98794	(−0.639, +0.549, −0.716)	77
**20**	12.90	12.04	12.61	1.66975	0.99577	(−0.624, +0.535, −0.700)	76
				1.71307	0.98607	(−0.621, +0.548, −0.718)	97
**21**	13.08	12.35	12.61	1.72608	0.99522	(−0.608, +0.538, −0.692)	84
				1.76602	0.98619	(−0.607, +0.552, −0.711)	104
**22**	12.71	12.09	12.56	1.71940	0.99448	(−0.631, +0.535, −0.694)	63
			12.63	1.75323	0.98626	(−0.631, +0.548, −0.712)	81
**23**	12.92	12.50	12.89	1.72271	0.99755	(−0.631, +0.535, −0.694)	63
				1.75509	0.98830	(−0.644, +0.549, −0.716)	72
**24**	13.15	12.45	13.00	1.67646	0.99547	(−0.627, +0.534, −0.700)	73
				1.71115	0.98643	(−0.626, +0.547, −0.717)	91
**25**	13.58	12.86	13.15	1.68007	0.99749	(−0.610, +0.535, −0.687)	77
				1.71520	0.98807	(−0.610, +0.549, −0.708)	98
**26**	13.38 13.91	12.30 12.95	13.43 13.98	1.66846	0.99736	(−0.622, +0.536, −0.676)	54
				1.72076	0.98570	(−0.617, +0.549, −0.699)	82
				1.63738	1.00210	(−0.610, +0.537, −0.701)	89
				1.68097	0.98996	(−0.606, +0.551, −0.721)	115
**27**	13.93	12.62	13.93	1.61806	1.00022	(−0.629, +0.536, −0.689)	60
			13.44	1.68256	0.98808	(−0.627, +0.550, −0.710)	83
**28**	14.12	13.28	14.11	1.65192	0.99990	(−0.621, +0.538, −0.696)	75
			13.99	1.69262	0.98997	(−0.621, +0.553, −0.716)	95
**29**	15.00	13.75	14.61	1.59292	1.00480	(−0.634, +0.539 −0.692)	58
				1.64796	0.99260		
**30**	15.20	14.03	14.94	1.58712	1.01611	(−0.630, +0.539, −0.686)	56
				1.63945	0.99403	(−0.631, +0.555, −0.708)	77
**31**	14.61 15.91	13.97 15.38	14.5	1.58832	1.00792	(−0.629, +0.538, −0.689)	60
				1.61450	0.99800	(−0.632, +0.553, −0.710)	78
			16.24	1.53351	1.01518	(−0.630, +0.539, −0.701)	71
				1.55290	1.00622	(−0.634, +0.555, −0.725)	91
**32**	16.76	16.49	17.09	1.5068	1.02233	(−0.629, +0.540, −0.692)	63
				1.51752	1.01495	(−0.635, +0.556, −0.717)	82
**33**	16.50	16.10	17.22	1.52904	1.02208	(−0.640, +0.538, −0.669)	29
				1.54311	1.01478	(−0.642, +0.554, −0.702)	60
**34**	17.20	18.05	17.3	1.46069	1.04128	(−0.693, +0.542, −0.696)	3
				1.44083	1.04060	(−0.707, +0.557, −0.723)	16
**35**	17.70	17.59	17.8	1.44455	1.04669	(−0.703, +0.542, −0.701)	−2
				1.40867	1.05278	(−0.722, +0.557, −0.729)	7
**Anions**							
**36**	14.34 ^a^(8.2%) ^c^ 14.25 ^b^(91.8%) 14.26 ^d^	16.82 ^a^(1%) ^c^ 14.61 ^b^(99%) ^c^ 14.63 ^d^	14.57	1.56178 ^a^	1.03503 ^a^	(−0.754, +0.541, −0.755)	−1
				1.6277 ^b^	1.01526 ^b^	(−0.758, +0.535, −0.748)	10
				1.50817 ^a^	1.04133 ^a^	(−0.772, +0.551, −0.781)	−9
				1.60161 ^b^	1.01290 ^b^	(−0.781, +0.548, −0.776)	5
**37**	19.32 ^a^(97.18%) ^c^	20.78 ^a^(46.01%) ^c^	19.36	1.38308	1.10012	(−0.793, +0.535, −0.762)	−31
	16.11 ^b^(2.82%) ^c^	17.32 ^b^(53.99%) ^c^		1.55928 ^b^	1.03303 ^b^	(−0.817, +0.540, −0.748) ^b^	−69
				1.29850	1.13567	(−0.810, +0.549, −0.792)	−18
	19.23 ^d^	18.91 ^d^		1.49879 ^b^	1.04125b	(−0.837, +0.551, −0.776) ^b^	−51
**38**	18.96 ^a^(11.87%) ^c^	20.29(1.0%)	19.37	1.37708 ^b^	1.10516	(−0.795, +0.538, −0.764)	−31
	16.30 ^b^(88.13%) ^c^	17.11 ^b^(99%) ^c^		1.53724 ^b^	1.03931 ^b^	(−0.808, +0.537, −0.755) ^b^	−53
				1.29212	1.14240	(−0.810, +0.550, −0.793)	−17
	16.62 ^d^	17.14 ^d^		1.48775 ^b^	1.04413 ^b^	(−0.827, +0.548, −0.784) ^b^	−43
**39**	20.25	22.11	19.90	1.40593	1.08017	(−0.743, +0.525, −0.764)	−22
				1.24084	1.13673	(−0.779, +0.540, −0.771)	−8
**40**	20.65	21.47	20.07	1.31074	1.13759	(−0.744, +0.530, −0.723)	−21
				1.23737	1.17881	(−0.764, +0.544, −0.758)	−6
**41**	19.35	20.47	20.27	1.40593	1.08017	(−0.792, +0.528, −0.762)	−30
				1.35039	1.09709	(−0.811, +0.543, −0.790)	−21
**42**	20.02	21.17	20.84	1.37790	1.08291	(−0.771, +0.526, −0.748)	−23
				1.31591	1.10506	(−0.790, +0.542, −0.774)	−16
**43**	22.77	22.89	21.33	1.19860	1.18586	(−0.746, +0.522, −0.747)	1
				1.19173	1.17940	(−0.772, +0.540, −0.770)	−2

^a^ Linear complex. ^b^ Bent complex. ^c^ Into parenthesis is the population (%) of the particular conformer. ^d^ Average chemical shift, δ_av_, taking into consideration the populations of the two low energy conformers.

**Table 2:** Comparison of the bond lengths involving hydrogen atoms of the X-ray, hydrogen-optimized and fully optimized structures of flurbiprofen. Reprinted, with permission, from [105]. Copyright 2005, The Royal Society of Chemistry.

Bond	Bond Length (Å)		
	X-ray	Calculated: Protons Relaxed	Calculated: All Atoms Relaxed
O–H	1.286	1.023	1.036
O···H	1.358	1.618	1.522
O–H···O	2.641	2.641	2.559
Methyl C–H	1.128	1.091	1.091
Aromatic C–H	1.132	1.083	1.084
C7–H	1.149	1.090	1.092

**Table 3:** Comparison of structural data of the calculated structures, at the B3LYP/6-31+G(d) (IEF-PCM in DMSO) and at the TPSS/TZVP (IEF-PCM in DMSO) level of theory, of the hypericinate (**1a**) with those of the X-ray structure [97]. Reprinted, with permission, from [50]. Copyright 2016, by Elsevier Science Ltd.

Interatomic Distances (Å)	Crystal Structure ^a^	B3LYP/6-31+G(d) (IEF-PCM in DMSO)	TPSS/TZVP (IEF-PCM in DMSO)
O(1)···O(14)	2.497(5)	2.526	2.497
O(13)···O(14)	2.494(4)	2.543	2.514
O(6)···O(7)	2.495(5)	2.528	2.497
O(8)···O(7)	2.529(5)	2.549	2.517
O(3)···O(4)	2.362(4)	2.384	2.365
O(1)–H(1)	0.89(6)	1.002	1.009
O(1)–H(1)···O(14)	1.74(6)	1.611	1.564
O(13)–H(13)	1.10(5)	1.001	1.006
O(13)–H(13)···O(14)	1.52(6)	1.633	1.591
O(6)–H(6)	1.13(6)	1.002	1.009
O(6)–H(6)···O(7)	1.50(6)	1.615	1.563
O(8)–H(8)	0.81(6)	1.000	1.007
O(8)–H(8)···O(7)	1.79(6)	1.643	1.585
O(3)···H(intra)	1.17(5)	1.081	1.125
O(3)–H(intra)···O(4)	1.20(5)	1.312	1.245
**Dihedral Angles (°)**			
C(10)–C(10a)–C(10b)–C(11)	32.4	34.0	33.9
C(3)–C(3a)–C(3b)–C(4)	19.2	19.8	18.5

^a^ Standard deviation in parenthesis.

## Supplementary Materials

Supplementary materials are available online.
