# Supplementary material for: Hydrogen Atomic Positions of O–H···O Hydrogen Bonds in Solution and in the Solid State: The Synergy of Quantum Chemical Calculations with 1H-NMR Chemical Shifts and X-ray Diffraction Methods
Source: Molecules. 2017 Mar 7;22(3):415. doi: 10.3390/molecules22030415 (PMC6155303; doi:10.3390/molecules22030415)
Supplement: Supplementary file 1 [file molecules-22-00415-s001.pdf]

Supplementary Material for Molecules

Hydrogen Atomic Positions of O–H···O Hydrogen Bonds in Solution and in the Solid State: the Synergy of Quantum Chemical Calculations with ^{1}H-NMR Chemical Shifts and X-ray Diffraction Methods

Michael G. Siskos[1], M. Iqbal Choudhary[2], Ioannis P. Gerothanassis[1,2*]

[1] Section of Organic Chemistry & Biochemistry, Department of Chemistry, University of Ioannina, Ioannina, GR-45110, Greece

[2] H.E.J. Research Institute of Chemistry, International Center for Biological and Chemical Sciences, University of Karachi, Karachi-75270, Pakistan

* Correspondence: igeroth@uoi.gr; Tel: +30 2651008389

Table S1. Calculated ^{1}H NMR chemical shifts (relative to TMS, ppm) of various hypericin + solvent (1:1 and 1:2) complexes with the GIAO/B3LYP/6-311+G(2d,p) – CPCM or IEF-PCM method. Reprinted, with permission, from [50]. Copyright 2016, by Elsevier Science Ltd.

Hypericin complex	method	C1 -OH	C6 -OH	(C1-OH + C6 –O)]/2	C13 -OH	C8 -OH	(C8 -OH+ :13 –OH)/2	C3 -OH	C4 -OH	(C3-OH + C4-OH)/2	C2 -H	C5-H	(C2 -H + C5-H)/2	C12 -H	C9-H	(C9-H + C12-H)/2	$C11-CH_3$	$C10-CH_3$	$(C10-CH_3 + C11-CH_3)/2$
HyH + acetone 1:1	B3LYP/6-31+G(d) (gas phase)	14.20	14.00	14.10	13.69	13.77	13.73	12.38	9.91	11.15	7.79	7.37	7.58	7.76	7.78	7.77	2.82	2.86	2.84
	B3LYP/6-31+G(d) (CPCM)	14.29	14.17	14.23	13.82	13.83	13.83	13.38	10.20	11.79	7.91	7.41	7.66	7.75	7.79	7.77	2.82	2.86	2.84
	TPSSh/TZVP (gas phase)	15.26	15.01	15.14	14.64	14.77	14.71	13.24	10.71	11.98	7.80	7.40	7.60	7.76	7.81	7.79	2.85	2.89	2.87
	TPSSh/TZVP CPCM	15.33	15.20	15.27	14.79	14.82	14.81	14.78	11.21	12.99	7.77	7.45	7.61	7.74	7.84	7.79	2.85	2.89	2.87
	CAM-B3LYP (CPCM)	14.13	14.01	14.07	13.66	13.68	13.67	13.62	10.23	11.92	7.85	7.31	7.58	7.65	7.68	7.66	2.73	2.77	2.75
HyH + acetone 1:2	B3LYP/6-31+G(d) (gas phase)	13.95	13.92	13.94	13.74	13.75	13.75	11.31	11.18	11.25	7.40	7.11	7.26	7.81	7.80	7.81	2.89	2.90	2.90
	B3LYP/6-31+G(d) (CPCM)	13.99	14.00	14.00	13.80	13.79	13.80	12.14	12.14	12.14	7.70	7.66	7.68	7.82	7.82	7.82	2.91	2.91	2.91
	TPSSh/TZVP (gas phase)	14.90	14.89	14.90	14.66	14.62	14.64	11.76	11.68	11.72	7.32	7.31	7.32	7.83	7.82	7.83	2.92	2.92	2.92
	TPSSh/TZVP (CPCM)	15.03	15.03	15.03	14.74	14.76	14.75	13.01	12.96	12.99	7.54	7.55	7.55	7.86	7.84	7.85	2.96	2.96	2.96
	CAM-B3LYP (CPCM)	13.90	13.90	13.90	13.69	13.69	13.69	12.29	12.29	12.29	7.58	7.61	7.59	7.70	7.70	7.70	2.84	2.84	2.84
HyH + DMSO 1:1	B3LYP/6-31+G(d) (gas phase)	14.15	14.02	14.09	13.69	13.78	13.74	13.22	10.15	11.69	7.50	7.36	7.43	7.84	7.77	7.81	2.87	2.90	2.89
	B3LYP/6-31+G(d) (IEF-PCM)	14.26	14.19	14.23	13.81	13.82	13.82	14.17	10.43	12.3	7.65	7.38	7.52	7.77	7.80	7.79	2.86	2.88	2.87
	TPSSh/TZVP (gas phase)	15.18	15.02	15.1	14.77	14.62	14.69	14.37	11.05	12.71	7.56	7.38	7.47	7.79	7.86	7.83	2.90	2.95	2.93

	TPSSh/TZVP (IEF-PCM)	15.29	15.22	15.26	14.80	14.76	14.78	15.78	11.57	13.68	7.72	7.39	7.56	7.79	7.81	7.80	2.89	2.91	2.90
	CAM-B3LYP (IEF-PCM)	14.19	14.13	14.16	13.75	13.76	13.76	14.43	10.55	12.49	7.70	7.37	7.54	7.74	7.79	7.77	2.85	2.87	2.86
	M062X/6-31G+d (IEF-PCM)	13.71	13.63	13.67	13.26	13.25	13.26	15.76	10.30	13.03	7.70	7.45	7.58	7.73	7.87	7.80	2.92	2.92	2.92
Hy^{-}	B3LYP/6-31+G(d) (gas phase)	14.33	14.24	14.29	14.30	14.44	14.37		19.70		6.99	6.88	6.94	7.68	7.68	7.68	2.78	2.78	2.78
	B3LYP/6-31+G(d) IEF-PCM (DMSO)	14.35	14.28	14.32	14.00	14.17	14.09		17.80		7.11	6.89	7.00	7.67	7.69	7.68	2.78	2.78	2.78
	TPSSh/TZVP (gas phase)	15.39	15.39	15.39	15.27	15.27	15.27		20.30		6.98	6.98	6.98	7.70	7.70	7.70	2.81	2.81	2.81
	TPSSh/TZVP IEF-PCM (DMSO)	15.37	15.31	15.34	15.01	15.11	15.06		19.26		7.09	6.98	7.04	7.71	7.71	7.71	2.79	2.81	2.80
	TPSSh/TZVP CPCM (DMSO)	15.37	15.31	15.34	15.01	15.11	15.06		19.25		7.09	6.98	7.04	7.71	7.71	7.71	2.79	2.81	2.80
	CAM-B3LYP IEF-PCM (DMSO)	14.17	14.22	14.20	13.85	14.04	13.95		17.65		6.77	7.00	6.88	7.60	7.60	7.60	2.69	2.69	2.69
	Crystal structure	12.91	11.80	12.36	19.34	12.18	15.76		20.03		2.48	4.24	3.36	5.59	4.86	5.23	-1.73	1.056	-0.34

Table S2. Results of the linear regression of calculated *vs* experimental ^{1}H-NMR chemical shifts determined from various minimized geometries of HyH and Hy^{-}[a] and the X-ray structure [97]. Reprinted, with permission, from [50]. Copyright 2016, by Elsevier Science Ltd.

Hypericin complex	Method	Correlation coefficient (R^2)	Mean square error	Slope
HyH + acetone 1:1	B3LYP/6-31+G(d) (gas phase)	0.9903 (0.9208)[b]	0.2707 (2.1175)[b]	1.0924 (1.0304)[b]
	B3LYP/6-31+G(d) (CPCM)	0.9917 (0.8926)[b]	0.2320 (2.8740)[b]	1.070 (0.9928)[b]
	TPSSh/TZVP (gas phase)	0.9966 (0.9167)[b]	0.0942 (2.2277)[b]	0.9888 (0.9276)[b]
	TPSSh/TZVP (CPCM)	0.9946 (0.871)[b]	0.1506 (3.4516)[b]	0.9589 (0.8777)[b]
	CAM-B3LYP (CPCM)	0.9901 (0.8771)[b]	0.2781 (3.2863)[b]	1.0701 (0.9852)[b]
HyH + acetone 1:2	B3LYP/6-31+G(d) (gas phase)	0.9943 (0.9146)[b]	0.1597 (2.284)[b]	1.0978 (1.0299)[b]
	B3LYP/6-31+G(d) (CPCM)	0.9982 (0.8673)[b]	0.3288 (3.5511)[b]	1.0788 (0.9885)[b]
	TPSSh/TZVP (gas phase)	0.9973 (0.9238)[b]	0.0763 (2.0393)[b]	1.0049 (0.946)[b]
	TPSSh/TZVP (CPCM)	0.9937 (0.8652)[b]	0.1753 (3.6063)[b]	0.9755 (0.8904)[b]
	CAM-B3LYP (CPCM)	0.9858 (0.8521)[b]	0.3977 (3.9557)[b]	1.0729 (0.9757)[b]
Hy^{-}	B3LYP/6-31+G(d) (gas phase)	0.9926	0.3324	0.9605
	B3LYP/6-31+G(d) (IEF-PCM in DMSO)	0.9981	0.0848	1.0561
	TPSSh/TZVP (gas phase)	0.998	0.0912	0.9079
	TPSSh/TZVP (IEF-PCM in DMSO)	0.9994	0.0271	0.9537
	CAM-B3LYP (IEF-PCM in DMSO)	0.9981	0.0833	1.0603
	X-ray crystal structure	0.9678	1.4428	0.7472

[a] The case of HyH +DMSO (1:1) was not examined since in DMSO solution hypericin exists in the ionic form (see text).
[b] The experimental value of δ = 8.2 ppm for the OH -3,4 protons of Smirnov *et al* .[99] was taken into account (see text).
